# Systematic identification of pan-cancer single-gene expression biomarkers in drug high-throughput screens

**DOI:** 10.1371/journal.pone.0330412

**Published:** 2026-05-11

**Authors:** Ginte Kutkaite, Göksu Avar, Diyuan Lu, Thomas J. O’Neill, Daniel Krappmann, Michael P. Menden

**Affiliations:** 1 Computational Health Center, Helmholtz Munich, Neuherberg, Germany; 2 Department of Biology, Ludwig-Maximilians University Munich, Martinsried, Germany; 3 Department of Biochemistry and Pharmacology, Bio21 Molecular Science and Biotechnology Institute, The University of Melbourne, Parkville, Victoria, Australia; 4 Research Unit Signaling and Translation, Molecular Targets and Therapeutics Center, Helmholtz Munich, Neuherberg, Germany; Weill Cornell University, UNITED STATES OF AMERICA

## Abstract

Precision oncology relies on molecular biomarkers to stratify patients into responders and non-responders to a given treatment. Although gene expression profiles have historically been explored for biomarker discovery, fewer studies investigated single-gene expression biomarkers. Additionally, many approaches are limited to cancer type-specific associations, which constrain statistical power. To address these limitations, we developed a regression-based framework that corrects for tissue-specific biases and enhances detection of pan-cancer single-gene expression biomarkers of drug sensitivity in cancer cell line high-throughput drug screens. Our method maintains predictive performance post-correction, and successfully recovers established biomarkers, such as *SLFN11* expression for DNA damaging agents. Notably, we identified *SPRY4* and *NES* expression as biomarkers of sensitivity for compounds targeting ERK/MAPK signaling (adjusted p-value = 4.016 × 10 ⁻ ⁵ and 7.221 × 10 ⁻ ⁶, respectively). This approach offers a scalable strategy for biomarker discovery and holds potential for translation to more complex biological models and patient-derived datasets. Ultimately, pan-cancer single-gene expression biomarkers may inform patient stratification and warrant clinical validation in precision oncology.

## Introduction

Precision oncology seeks to improve treatment outcomes by stratifying patients based on their molecular profiles to predict therapeutic response [1]. Despite advances in molecular profiling technologies, drug development remains high-risk, with clinical trial failure rates nearing 95% [2, 3] often due to the absence of reliable biomarkers for identifying responsive subgroups. This underscores the urgent need for novel biomarkers and innovative application strategies to accelerate drug development and improve clinical success [1].

Biomarker discovery remains a major challenge in precision oncology. Large-scale efforts such as The Cancer Genome Atlas (TCGA) [4] and the International Cancer Genome Consortium (ICGC) [5] have mapped tumor molecular profiles, but largely lack linked treatment records and clinical outcomes. Real-world data (RWD) sources like Flatiron Health [6] integrate molecular and clinical data from hospital cohorts but are limited by sparse coverage of investigational therapies, non-randomized treatment assignment, variable data quality, and restricted accessibility.

High-throughput drug screens in molecularly profiled cancer cell lines offer a scalable framework for biomarker discovery. Pioneering efforts such as the NCI-60 screen [7] laid the foundation for larger-scale resources, including the Genomics of Drug Sensitivity in Cancer (GDSC) [8, 9] and the Cancer Cell Line Encyclopedia (CCLE) [10], which profile drug responses in over 1,000 cancer cell lines spanning diverse tissue types. The integration of these data with multi-omics characterization supports the identification of pan-cancer biomarkers and mechanistic insights across genomic, transcriptomic, and epigenetic layers [9, 11–13].

Pan-cancer pharmacogenomic approaches leverage the diversity of cancer cell lines to identify biomarkers that generalize across tumor types. By pooling molecular and drug response data beyond a single lineage, such analyses increase statistical power and can reveal mechanisms shared across distinct tumor contexts [14, 15]. However, this design also introduces strong tissue-of-origin confounding, since gene expression is highly structured by lineage and histotype [14–17]. Correcting for these biases is critical to distinguish true pan-cancer signals from spurious lineage-driven effects, thereby improving both biological interpretability and cross-dataset transferability.

A wide range of statistical and machine learning (ML) frameworks have been developed to model drug response in cancer cell lines, with varying trade-offs between predictive performance and interpretability. Biomarker discovery approaches span from univariate ANOVA models [9, 11] to multivariate regularized linear regression [12]. While more complex ML models, such as support vector machines, random forests, and deep neural networks, may offer higher predictive accuracy [18–20], they often lack interpretability. To improve interpretability, post hoc model-agnostic methods such as Shapley values [21] and LIME [22] have been developed to quantify how individual features influence model predictions. Recent GDSC-based studies have applied interpretable and integrative modeling to predict drug response, integrating pharmacogenomic and patient transcriptomic data [23–25], yet these primarily capture global transcriptomic patterns rather than systematic, per-drug single-gene biomarkers.

Cancer is driven by genetic alterations, and accordingly, most drug response biomarkers are based on mutations, copy-number changes, or structural variants [26]. As one of the earliest and most extensively characterized molecular layers, genomics has yielded numerous mutation-based biomarkers across various cancer types [27, 28]. While other omics layers, such as transcriptomics and proteomics, have also been widely investigated, their integration into systematic biomarker discovery efforts has been comparatively limited [27, 28]. Gene expression (GEX) signatures, also referred to as endotypes, are increasingly recognized for their association with drug response and are beginning to enter clinical practice [27, 29]. However, single-GEX biomarkers are comparatively rare, partly due to their transient and context-dependent nature. A notable exception is *SLFN11*, whose upregulation sensitizes cancer cells to DNA-damaging agents and has been validated in preclinical models [10, 30–32].

GEX exhibits strong tissue-of-origin dependency, representing a major obstacle for the identification of pan-cancer biomarkers [14, 16, 17]. As a transient omic layer, it is governed by tissue-specific regulatory programs, leading to high consistency within tissues but poor comparability across them [33]. This tissue effect can confound associations with drug response, obscuring signals that generalize across cancer types. Consequently, many studies are restricted to single-tissue analyses, which limits statistical power and prevents leveraging cross-tissue or transfer learning opportunities.

Here, we identify pan-cancer single-GEX biomarkers predictive of drug sensitivity. To reduce tissue-specific bias, we implemented two correction strategies: (1) z-score normalization and (2) residual adjustment. We then applied regularized linear regression to associate corrected single-GEX with drug response across cancer cell lines. Focusing on individual genes enables interpretable models, while the pan-cancer design increases sample size, statistical power, and transfer learning across cancer types. We hypothesize that this approach will recover known drug targets and uncover novel, clinically relevant biomarkers.

## Results

**Fig 1:**
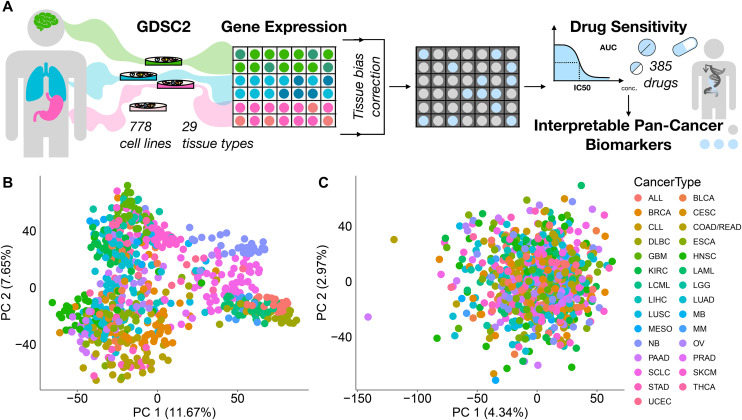
Tissue type dependencies in cancer cell line gene expression data. **(A)** Analysis workflow to identify gene expression biomarkers. **(B)** Principal Component Analysis (PCA) plot depicting gene expression data coloured by the cancer cell line tissue of origin. **(C)** PCA plot showing z-score corrected gene expression data.

For the discovery of pan-cancer single-gene expression (GEX) biomarkers, we first addressed tissue-of-origin effects in cancer cell lines. We analyzed GDSC data comprising 778 cell lines across 29 cancer types and drug response to 385 compounds targeting 24 pathways (Fig 1A), with response quantified as area under the curve (AUC). Principal component analysis revealed strong tissue-specific expression patterns, particularly between solid and non-solid tumors (Fig 1B; S1 FigA). To mitigate this, we applied z-score normalization and residual-based correction (**Methods**). Post-correction, tissue-specific clustering was no longer evident (Fig 1C; S1 FigB-D), enabling a more robust and unbiased identification of pan-cancer expression biomarkers.

### Disentangling tissue effects improves the accuracy of pan-cancer drug response predictions

**Fig 2:**
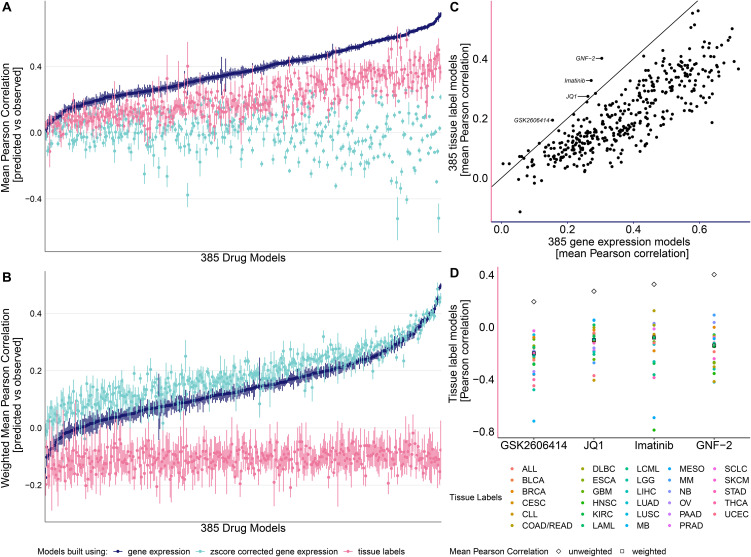
Performance of 385 drug models built using gene expression, z-score corrected gene expression and tissue labels. **(A)** Unweighted and (**B**) weighted Pearson correlation of 385 drug response models either leveraging gene expression, z-score corrected gene expression or tissue labels. **(C)** Mean unweighted Pearson correlation of drug models using tissue labels and gene expression. **(D)** Pearson correlation within individual tissue types as well as mean unweighted and weighted Pearson correlation.

To systematically predict drug response across the 385 compounds in a pan-cancer setting, we evaluated regularized linear regression models. While ridge regression underperformed relative to lasso and elastic net when using tissue labels as input, it outperformed both methods with GEX data (Wilcoxon signed-rank test adjusted p-value = 2.28 × 10 ⁻ ⁶⁴ vs. Lasso; 2.27 × 10 ⁻ ⁶⁴ vs. Elastic Net; estimate = 0.063; S2 FigA-D), and was therefore selected for all subsequent analyses (S1 Table). We trained 1,155 drug-specific ridge models using three input types: tissue labels (naïve baseline), uncorrected GEX (tissue-confounded), and tissue-corrected GEX (**Methods**; Fig 2A).

A key challenge is Simpson’s paradox, arising from strong tissue-specific drug responses, for instance, cell lines derived from non-solid tumors often require lower drug concentrations than solid tumors, inflating prediction-observation correlations (S3 Fig). Without bias correction, tissue labels and uncorrected GEX appear highly predictive (Fig 2A; S2 FigE). Consequently, tissue-corrected GEX models seem to underperform (p-value<2.2 × 10 ⁻ ¹⁶; pseudo-median = −0.358 vs. GEX, pseudo-median = −0.197 vs. tissue; Fig 2A), although this reflects non-translatable tissue effects with limited clinical utility.

To account for confounding due to tissue-specific drug responses and imbalanced tissue representation, we evaluated model performance using tissue-weighted Pearson correlation (**Methods;** Fig 2B). This adjustment effectively removed the predictive advantage of models relying solely on tissue labels, which subsequently performed at random or at overfitted levels (Fig 2B; S2 FigF). Notably, uncorrected GEX retained most its predictive power, but was significantly outperformed by tissue-corrected GEX models (p-value<2.2 × 10 ⁻ ¹⁶, pseudo-median = 0.048), highlighting their value for pan-cancer drug response prediction (Fig 2B; S4 Table).

Gene expression (GEX) encodes both tissue-of-origin and additional mechanistic information [34], evidenced by GEX-based models generally outperforming tissue-based models in predicting drug response (Fig 2A). We identified 10 exceptions where tissue labels yielded better performance (Fig 2C), with four models exceeding a correlation of 0.15. These cases suggest that tissue origin may act as a proxy biomarker, and GEX models may overfit without adding mechanistic insight. Notably, certain cancer types are defined by genetic alterations; for example, imatinib and GNF-2, which target ABL, performed best in BCR-ABL-positive tissues characteristic of chronic myeloid leukemia (CML) [35]. However, this association is highly tissue-dependent and entirely lost within cancer type context (Fig 2D; S2 FigG-J). These findings underscore the importance of not relying solely on the tissue context when refining predictive modelling and guiding biomarker discovery.

### Feature extraction allows pan-cancer gene expression signature discovery

Patient stratification in clinical settings necessitates the extraction of interpretable biomarkers of drug response. Here, we focused on predictive models derived from cancer cell line data, which serve as a preclinical framework for identifying such biomarkers. Ensuring robust model performance is essential to guarantee that selected features reflect meaningful biological signals. To systematically identify such models, we constructed null models and applied standard deviation-based thresholding, yielding 266 informative drug models spanning 23 distinct pathways (**Methods;** S4 Fig).

**Fig 3:**
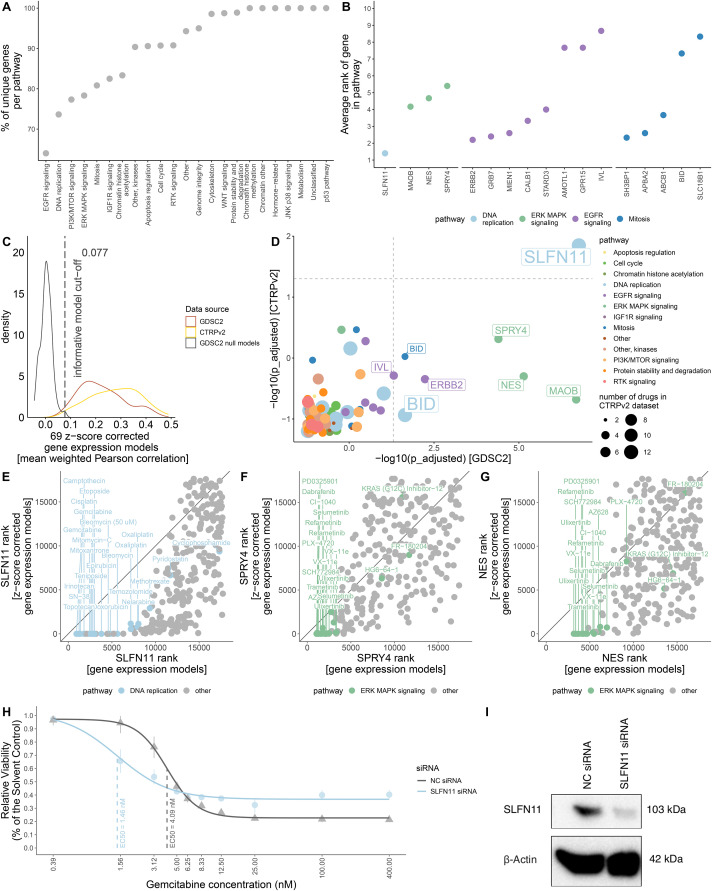
Robust pan-cancer biomarker selection. **(A)** The percentage of unique genes per pathway; (**B**) the average rank of genes for drugs in a specific pathway; (**C**) distribution of performance of 69 drug models built using the GDSC and the CTRP datasets; (**D**) adjusted p-values from overrepresentation tests with the GDSC and the CTRP datasets (points with negative enrichment score coloured by pathway); the rank of **(E)** *SLFN11*, **(F)** *SPRY4,* and **(G)** *NES* in models built with gene expression and z-score corrected gene expression inputs. **(H)** Dose-response curves of A375 melanoma cells transfected with *SLFN11* and negative control (NC) siRNAs and treated with gemcitabine for 72 hours. Dots and whiskers represent the mean viabilities with 95% confidence interval. **(I)** Western blot showing the decrease in SLFN11 protein levels 72 hours after transfection with siRNA.

Recurrent biomarkers shared across drugs targeting the same pathway may reflect underlying mechanistic associations. We investigated this by analyzing feature overlap across the 266 informative models at the pathway level (Fig 3A**; Methods**). Models targeting EGFR signaling exhibited the fewest unique top-ranked features, suggesting strong recurrence of specific genes among the top 10 features across these drugs. To quantify this, we identified features that appeared in the top 10 for at least 25% of drugs targeting the same pathway (Fig 3B), highlighting candidates with potential pathway-level relevance.

Independent validation is essential to assess the robustness and translational potential of identified biomarkers. To this end, we used the Cancer Therapeutics Response Portal (CTRP) dataset, which provided matching drug response and GEX data for 69 of the 266 informative drugs identified in GDSC (S5 Fig; S2 Table). Model performance showed significant concordance between the two datasets, particularly for tissue-corrected GEX models (Wilcoxon signed-rank test: GDSC vs. CTRP, p-value = 9.688 × 10 ⁻ ⁵, pseudo-median = −0.048; GDSC null vs. CTRP, p-value = 5.331 × 10 ⁻ ¹³, pseudo-median = −0.275; Fig 3C). Similar trends were observed in the smaller, historical NCI-60 panel [7, 36], where z-score correction mitigated tissue effects and *SLFN11* remained a consistent predictor following short (2-day) and long (11-day) drug exposures (**Methods**; S6 Fig), further supporting the reproducibility of our modeling framework across an independent dataset.

To systematically assess pathway-level biomarker recurrence, we performed hypergeometric enrichment analysis to identify genes consistently ranked among the top features in drugs targeting the same pathway (**Methods**). This analysis confirmed *SLFN11*, a well-established and widely validated biomarker for sensitivity to DNA-damaging agents, as a recurrent feature in DNA replication-targeting drugs across both datasets (Fig 3D; S7 FigB-C), serving as a positive control that supports the validity of our approach. Although the smaller sample size in CTRP (S5 FigC-D) limited replication of all top associations from GDSC, it nevertheless enabled the recovery of key biomarkers with consistent trends (Fig 3D), yielding a set of candidates for further investigation.

In support of this, *SLFN11* was selected in 68% of DNA replication-targeting models (S7 FigA), with an average rank of 1.4 using z-score-corrected GEX (Fig 3B). It was significantly overrepresented in both datasets following tissue bias correction (ES GDSC = −0.932, adjusted p-value = 1.718 × 10 ⁻ ⁷; ES CTRPv2 = −0.892, adjusted p-value = 0.014; Fig 3D-E; S7 FigB-C), further supporting the robustness and reproducibility of our findings.

We next examined recurrent gene expression biomarkers associated with other drug-targeted pathways beyond DNA replication. Within the ERK MAPK signaling pathway, *SPRY4* and *NES* emerged as notable candidates. *SPRY4* and *NES* were selected in 28% and 33% of models, respectively, with average ranks of 5.4 and 4.7 (S7 FigA; Fig 3B). Both genes were significantly overrepresented in the GDSC dataset after tissue correction (*SPRY4*: ES = −0.911, adjusted p-value = 4.016 × 10 ⁻ ⁵; *NES*: ES = −0.908, adjusted p-value = 7.221 × 10 ⁻ ⁶; Fig 3D, F,G; S7 FigB), suggesting their potential as pathway-specific biomarkers.

We also recovered *ERBB2* in models targeting EGFR signaling, consistent with its well-established role as a biomarker supported by multiple studies [37–40] (Fig 3B, D; S7 FigA-C, E). In addition, other promising candidates emerged, including *MAOB* for ERK MAPK signaling, *IVL* for EGFR signaling, and *BID* for mitosis and DNA replication-targeting drugs (Fig 3B, D; S7 Fig). Supporting this association, *BID* expression was significantly higher in paclitaxel responders than in non-responders in the I-SPY2 neoadjuvant trial (Welch’s t-test p-value = 0.019; **Methods;** S7 FigJ), aligning with its inferred role in mitosis-targeting drug sensitivity. These findings highlight a broader spectrum of gene expression biomarkers that may warrant further functional validation and investigation.

We next assessed how tissue correction affected the composition and tissue dependence of top-ranked gene features across the 266 informative models (**Methods;** S8 Fig). Emerged features, detected only after correction, dominated (94.7%), whereas retained features, shared between both models, accounted for 5.3% of all genes appearing in the combined top 10 sets (S8 FigA; S5 Table). Within the top 10 features, retained genes displayed lower tissue attribution (median $\eta^{\text{2}}$ = 0.092) than emerged genes (median $\eta^{\text{2}}$= 0.223) (S8 FigB; S6 Table). All four biomarker candidates, SLFN11, NES, SPRY4, and ERBB2, belonged to the emerged class and showed improved ranks after correction (e.g., SLFN11 median delta rank=+6078; S8 FigC-F). Together, these results indicate that tissue correction improves feature stability and prioritizes biomarkers with cross-tissue predictive relevance, motivating further evaluation of their tissue-specific associations with drug sensitivity.

To further refine their context-of-use, we correlated uncorrected gene expression with drug sensitivity across cancer types (**Methods**; S7 Table). *SLFN11* expression was strongly associated with gemcitabine response in glioblastoma (Pearson r = −0.84, adjusted p-value = 5.049 × 10 ⁻ ^4^) and remained significant in additional tumor types, consistent with its broad role in DNA-damage response (S9 Fig). *ERBB2* expression correlated with osimertinib response in lung squamous cell carcinoma (Pearson r = −0.90, adjusted p-value = 6.704 × 10 ⁻ ^3^; S10 Fig) and showed similar patterns across other cancers. *SPRY4*, *NES*, *IVL*, *MAOB*, and *BID*, also exhibited lineage-dependent associations with drugs targeting their respective pathways (S11**-**S15 Fig). These findings support the translational relevance of the identified biomarkers by linking them to specific cancer contexts.

Finally, we sought experimental validation of *SLFN11* as a gold standard biomarker to confirm the reliability of our computational framework. Given its well-established role in sensitizing cells to DNA-damaging agents, as well as consistent associations across GDSC and CTRP datasets, *SLFN11* was prioritized for *in vitro* validation over newly identified candidates. In *SLFN11*-knockdown A375 melanoma cells treated with gemcitabine, a DNA replication-targeting drug, we observed reduced drug efficacy upon *SLFN11* downregulation (EC50 NC = 4.09nM vs EC50 SLFN11 = 1.46nM; Fig 3H-I; S7 FigI). This result not only aligns with known biology but also demonstrates that our framework can identify biomarkers with strong mechanistic and translational relevance.

## Discussion

Genomic profiling within individual cancer types has driven early success in precision oncology by enabling targeted therapies against recurrent oncogenic mutations. However, progress has slowed due to tumor heterogeneity, limited cohort sizes, and the rarity of actionable mutations, all of which constrain predictive modeling and clinical translation. In contrast, gene expression (GEX) profiling and pan-cancer analyses remain underutilized [27–29, 41], despite their potential to capture functional tumor states and offer increased statistical power. Harnessing these complementary data layers presents a key opportunity to accelerate progress in precision oncology.

Cancer cell lines offer a scalable model for drug response studies, enabling experiments not feasible in patient-derived samples. Large-scale screens such as NCI-60, GDSC, and CTRP have validated known biomarkers and identified novel ones using statistical and machine learning methods [7, 9–13]. Tissue-specific models often miss biomarkers in rare cancer types due to limited sample representation [9, 11, 12]. Pan-cancer approaches improve predictive performance but may obscure biological mechanisms, as they group distinct diseases that, despite shared hallmarks, differ in molecular pathogenesis [18–20, 42, 43].

This study advances current computational approaches by systematically leveraging gene expression data in cancer cell lines to identify robust pan-cancer single-gene biomarkers. Our framework enables deeper insights into drug mechanisms of action and provides a scalable basis for hypothesis generation with translational potential, pending validation in patient-derived models and clinical cohorts. Notably, models incorporating tissue type-corrected gene expression retain strong predictive performance while yielding biologically interpretable biomarkers (Fig 2B; Fig 3D). However, not all drug-biomarker associations are expected to generalize across tissues, and several limitations need to be considered.

The generalizability of pan-cancer biomarkers is constrained by several biological and modeling limitations. Lineage-specific oncogene dependencies exemplify cases where therapeutic response is restricted to particular cellular contexts and would not emerge as cross-tissue expression biomarkers; a canonical example is the activity of BCR-ABL inhibitors in chronic myeloid leukemia [35, 44, 45]. Some predictive associations are also primarily encoded in other molecular layers, such as mutations, fusions, or copy-number alterations, rather than baseline transcript levels, and therefore remain inaccessible to single-gene expression models. Furthermore, cancer cell line systems lack key components of the tumor microenvironment, including stromal, immune, and metabolic interactions. These interactions are known to influence therapeutic response and their absence limits the biological scope of detectable signals, representing a further barrier to direct clinical translation [46–49]. Moreover, treatment-induced transcriptomic changes are not captured by basal profiling, representing a complementary pharmacodynamic dimension accessible through resources such as LINCS L1000 [50, 51]. Together, these constraints define the boundaries within which pan-cancer single-gene expression biomarkers can be identified and interpreted.

While biological and modelling constraints limit the scope of generalizable biomarkers, effective correction of tissue-driven confounding remains essential for identifying meaningful pan-cancer signals from gene expression data. We evaluated two correction strategies: residual-based and z-score normalization. Both approaches reduced tissue-driven variation, but z-score normalization provided a more stringent correction (Fig 1C; S1 FigB-D). In contrast, residual-based correction retained subtle tissue-specific signals, as reflected in residual clustering when comparing solid vs non-solid tumor types (S1 FigD). Residual correction may nonetheless be useful in contexts where biomarkers are expected to be partly modulated by tissue lineage, whereas z-score normalization is better suited for identifying generalizable pan-cancer signals. Based on these observations, we used models trained on z-score normalized expression data for downstream biomarker interpretation.

The choice of modeling framework represents another key consideration in large-scale pharmacogenomic analyses. We employed regularized linear regression, which provides robust performance and direct interpretability of gene-level coefficients across thousands of predictors and compounds [9, 11, 52–54]. Although linear models do not explicitly capture nonlinear dependencies or hierarchical variance components, they enable transparent feature attribution and reproducible identification of single-gene biomarkers [55–58]. Deep learning approaches, while capable of modeling complex nonlinear relationships, are susceptible to overfitting given the high feature-to-sample ratio inherent to transcriptomic datasets of this scale and require post-hoc methods to recover feature-level interpretability [18, 19, 59, 60]. Alternative approaches, such as mixed-effects or hierarchical models, could more formally account for tissue-nested variance and lineage-drug interactions; however, their computational demands and reduced interpretability limit their scalability in pan-cancer settings [61–63]. Future integration of such hybrid strategies with regularized regression frameworks may further refine tissue correction and improve the modeling of cross-lineage heterogeneity.

Robust biomarker discovery should recover established associations and reveal biologically plausible candidates across diverse drug classes. In our analysis, the strongest biomarker signals were observed for compounds targeting DNA replication, ERK MAPK signaling, EGFR signaling, and mitosis (Fig 3B). As expected, we recapitulated well-characterized biomarkers, including *ERBB2* for EGFR-targeting agents [37–40] and *SLFN11* for DNA replication inhibitors [10, 30–32, 64–66]. Consistent with this, siRNA-mediated *SLFN11* downregulation in A375 melanoma cells reduced gemcitabine efficacy (Fig 3H-I), providing experimental support for the framework’s ability to identify biomarkers with mechanistic and translational relevance. Supporting the broader translational potential of the identified biomarkers, exploratory analysis of the I-SPY2 neoadjuvant trial (NCT01042379) [67, 68] suggested a potential link between *BID* expression and paclitaxel sensitivity (S7 FigJ), consistent with its inferred role in mitosis-targeting drug response.

ERK/MAPK pathway activity emerged as a key determinant of drug response in our analysis (Fig 3B). Expression of *SPRY4* and *NES* correlated with sensitivity to ERK/MAPK pathway inhibitors. *SPRY4* encodes a known negative regulator of MAPK signaling via inhibition of GTP-bound RAS formation [69–71]. Loss of SPRY4 has been associated with invasive phenotypes, consistent with a role in modulating MAPK-dependent cellular states that influence sensitivity to pathway inhibition [72, 73]. Although *SPRY4* has not previously been reported as a single-gene biomarker, it contributes to the MAPK Pathway Activity Score (MPAS), a transcriptional signature predictive of MEK1/2 inhibitor response in multiple cancer types [74]. Our findings therefore support *SPRY4* expression as a potential surrogate marker of ERK/MAPK pathway activity and drug sensitivity.

*NES* serves as an additional gene expression biomarker of sensitivity to ERK/MAPK pathway inhibitors. NES is an intermediate filament protein that facilitates mitotic progression through disassembly of phosphorylated vimentin [75–77]. It is recognized as a cancer stem cell marker [78] and promotes tumor proliferation and invasion via mitochondrial remodeling [79]. Supporting our findings, *NES* expression in melanoma has been linked to increased sensitivity to BRAF and MEK inhibitors, including dabrafenib and trametinib [80]. Consistent with this, reduced *NES* expression has been associated with acquired resistance to MAPK pathway inhibition, accompanied by increased proliferation, invasiveness, and activation of integrin and PI3K/AKT/mTOR signaling, supporting a role in adaptive drug response [81]. To place these findings in a broader translational context, we next consider how this biomarker discovery aligns with the principles of predictive, preventive, and personalized medicine.

The framework of Predictive-Preventive-Personalized Medicine (PPPM) emphasizes three complementary aims: prediction of therapeutic response, prevention through risk stratification and early detection, and personalization of treatment [82, 83]. Our study primarily advances the predictive dimension by identifying single-gene expression biomarkers with cross-dataset concordance and experimental support (e.g., SLFN11). In a preventive context, these biomarkers generate hypotheses for stratifying patients into higher- or lower-risk groups and for integrating expression signals with imaging or circulating biomarkers to detect emerging resistance. Future validation in patient cohorts will be essential to assess the prognostic and treatment-predictive value of biomarkers such as SPRY4 and NES and to evaluate their integration with clinicopathological and molecular features to inform PPPM-guided treatment strategies.

Translating pan-cancer biomarkers into clinical practice requires validation in systems of progressively increasing biological complexity. Patient-derived organoids and xenograft models better preserve tumor architecture, lineage context, and microenvironmental signaling dependencies, and represent the most immediate next step for the biomarkers identified here [47, 84, 85]. Biomarker-drug associations may exhibit marked tissue specificity (S9**-**S15 Fig), underscoring the importance of lineage-stratified validation and the need to account for tissue-specific effects during clinical translation [15, 86]. For SLFN11 specifically, studies integrating immunohistochemistry and transcriptomic profiling have shown discrepancies between tumor-intrinsic and bulk RNA-seq estimates, highlighting the need for IHC or RNA-ISH assays to capture clinically relevant expression levels [86, 87]. Together, these considerations outline a practical path for advancing biomarker discovery from preclinical models toward clinical implementation.

In conclusion, our pan-cancer gene expression analysis of cancer cell lines identified both known and novel drug sensitivity biomarkers, including SPRY4 and NES for ERK/MAPK pathway inhibitors. This approach offers a scalable framework for generating biomarker hypotheses across diverse drug classes and can be readily extended to other preclinical models, including patient-derived organoids and xenografts, to better capture tumor heterogeneity and improve clinical translatability. Positioned within the framework of predictive, preventive, and personalized medicine, such integrative analyses may ultimately inform the development of systematic, biomarker-guided strategies for tailored treatment selection in oncology.

## Materials and methods

### Cancer cell line characterization

Robust Multichip Average (RMA) normalized basal gene expression data as well as annotations such as MSI status, growth properties and culture media information for 1,001 cell lines can be downloaded from GDSC portal (https://www.cancerrxgene.org/downloads).

From 1,001 cell lines, 223 were filtered out as they did not have full information, namely, gene expression, consistent tissue labels or drug response data. Therefore, models were built on 778 cancer cell lines (see S3 Table for cancer cell line counts per cancer type).

### Drug response data

The drug response data can be downloaded from the Genomics of Drug Sensitivity in Cancer (GDSC) portal data release 8.0 (https://www.cancerrxgene.org/downloads). Where available, we have used GDSC2 data. Drug response was quantified by area under the drug response curve (AUC).

Out of 400 tested drugs 15 were filtered out where a given drug was tested in fewer than 10% of all cell lines (n=778) or tested in only one specific cancer type, leaving 385 drugs to be used in building pan-cancer models. To provide context on assay coverage and tissue representation, S3 Table (counts_drug sheet) reports the number of screened cell lines per tissue type for each drug.

### Cancer Therapeutics Response Portal (CTRP) validation data

For validation of our pan-cancer biomarkers, we have used drug response (AUCs) and basal gene expression data downloaded from CTRPv2 via National Cancer Institute portal (https://ctd2-data.nci.nih.gov/Public/Broad/CTRPv2.1_2016_pub_NatChemBiol_12_109/). Out of 481 drugs in the dataset, 69 were overlapping with our informative drugs (**Methods**; S5 FigC) and had corresponding gene expression information. Models for these drugs were built using gene expression and z-score corrected gene expression matrices (**Methods**) from 822 cell lines across 23 cancer lineages (S5 FigA-B).

### Drug response predictions

To predict drug response and ultimately retrieve pan-cancer biomarkers, we have employed linear regression models, namely ridge [53], lasso [52] and elasticNet [54], from glmnet R package. The fundamental difference between these models is the values of the tuning parameter alpha (0<α<1). with Ridge defined by α=0 and Lasso defined by α=1. For elasticNet we have tested alphas of 0.2, 0.4, 0.5, 0.6 and 0.8 with tissue label as well as gene expression matrices as input (S2 FigB, D). No significant difference in model performances was noted between different alpha parameters.

For all models, 10-fold cross-validation is applied and repeated 10 times. The weighted and unweighted Pearson correlations were used to evaluate the model performance. The weighted Pearson correlation ($p_{w}$) was calculated as follows,


$$p_{w}=tanh\left(\frac{\sum_{i=\text{1}}^{N}\sqrt{n_{i}-\text{1}}\;arctanh\left(p_{i}\right)}{\sum_{i=\text{1}}^{N}\sqrt{n_{i}-\text{1}}}\right),$$

where i is an individual cancer type, N=29 is the number of tested cancer types and $p_{i}$ is an unweighted Pearson correlation. For a given tissue type and drug combination, at least 3 cell lines had to be treated ($n_{i}\geq \text{3}$).

### Z-Score-based tissue-correction

To account for the difference between tissues, gene expression data is normalized by subtracting the tissue-specific mean and divided by the tissue-specific standard deviation. The z-score is calculated as shown below


$$Z=\frac{x-\mu_{i}}{\sigma_{i}},$$

where $\mu_{i}$ and $\sigma_{i}$ are the mean and standard deviation of tissue type i (i=29) across all cell lines, respectively. x is a RMA normalized gene expression count. Unless specified otherwise, all results reported to tissue-corrected GEX are based on the z-score correction.

### Residual-based tissue-correction

Additionally to the z-score-based correction, we built generalized linear models to predict gene expression profiles from the tissue type labels alone, followed by residual extraction. The procedure was repeated ten times and an averaged residual matrix was subsequently used to predict drug dose response in pan-cancer. Yielding similar results to z-score based correction, these results were reported in the supplements (S1 FigC-D, S4 FigC, and S2 Table).

### Null models and thresholding

In order to select informative models, a suitable performance threshold is needed. To select this threshold, we have built null models with shuffled drug dose-response data. For each drug model (n=385), we have generated ten null-matrices, which serve as the drug response baseline for predictions.

A distribution of null models with mean-weighted Pearson correlation values was built. The performance threshold is defined as the mean plus three standard errors of the null model distribution, a conservative criterion consistent with a normal approximation. This was used to select informative models for each input matrix, namely, tissue labels, gene expression, residual- and z-score-corrected gene expression (S4 FigA-D). We have annotated 266 models as overall informative (S4 FigE-F).

### Feature selection and processing

To better understand the biological implication, we have further investigated the features of those selected informative models (n=266). In this context, features denote the genes (n=17,737) used as input variables in the gene-expression-based models. Consider the total number of genes is G. For drug d, 10 independent runs were performed and the weights for each gene g in all 10 runs were collected and averaged, denoted by ${\overset{―}{w}}_{dg}$. Then, the averaged weight ${\overset{―}{w}}_{dg}$ (g=1,…,G) was sorted by their absolute values in a descending manner across all genes. This gave rise to the average rank of gene g for drug d, denoted by ${\overset{―}{r}}_{dg}=(\text{1},\ldots ,G)$, which is the index of ${\overset{―}{w}}_{dg}$ in the sorted weight vector. This was repeated for all the informative drug models (n=266).

To summarize feature information on a pathway level, we focused on those drug models that target the same pathway. Given a total number of $d_{p}$ drug models targeting pathway p, we only considered the top 10 ranked genes in each drug model, i.e.,  ${\overset{―}{r}}_{dg}=(\text{1},\ldots ,\text{1}\text{0})$ , resulting in a total number of $d_{p}\times \text{1}\text{0}$ genes (one gene might appear multiple times). Then, the percentage of the unique genes for pathway p was computed by


$$\left(\frac{n_{\text{unique genes}|p}}{d_{p}\times \text{1}\text{0}}\right)\times \text{1}\text{0}\text{0},$$

where $n_{\text{unique genes}|p}$ is the number of unique genes for pathway p.

### Assessment of feature stability and tissue attribution

To extend the feature analysis, we next examined how tissue correction influences the composition and tissue dependence of top-ranked genes across informative drug models (n=266). We evaluated the effect of tissue correction on feature selection using the top 10 ranked genes from each model trained on raw and tissue-z-score-corrected gene expression matrices. We classified genes as retained when present in both raw and corrected models, and as emerged when present only after correction. We calculated the proportions of retained and emerged features within the union of top 10 gene sets. We mapped drugs to pathway annotations and summarized mean proportions per pathway, reporting the number of contributing models per pathway (S5 Table).

We quantified tissue attribution for gene expression using the proportion of variance explained by tissue of origin ($\eta^{\text{2}}$). For each gene, we calculated between-tissue and total sums of squares across cancer types and defined


$$\eta^{\text{2}}=\frac{SS_{\text{between}}}{SS_{\text{total}}}$$

We summarized the distribution of $\eta^{\text{2}}$ across all genes (S8 FigB; S6 Table) and compared values between retained and emerged feature classes by joining $\eta^{\text{2}}$ to the top 10 union membership.

We assessed changes in feature ranking following tissue correction by computing


$$\Delta \text{rank}={\text{rank}}_{\text{exp}}-{\text{rank}}_{\text{zscore}},$$

where positive values indicate improved rank after correction. We summarized Δrank distributions for selected biomarker genes (SLFN11, NES, SPRY4, and ERBB2) (S8 FigC-F).

### Hypergeometric enrichment analysis

We ran an enrichment analysis to systematically identify which features are overrepresented with high ranks in certain drug pathways. To this end, we leveraged the fgsea function from fgsea R package with a vector of drugs ranked from lowest to highest weight for each feature of interest (n=17, selected from Fig 3B). Here, only pathways targeted by at least two drugs were considered. The enrichment scores (ES), p-values as well as the Bonferroni p-adjusted values were estimated for each feature and pathway combination.

### NCI-60 validation dataset

Baseline gene expression data for the NCI-60 cancer cell line panel were obtained from the CellMiner database (dataset “xai”, average log₂ intensity across Affymetrix platforms), and drug response (IC50) data were retrieved from the National Cancer Institute Developmental Therapeutics Program (https://brb.nci.nih.gov/ETvsCT/) [7, 36, 88]. Expression data were filtered to retain tissues represented by at least three cell lines and were used either in raw form or z-score normalized within tissue, as described above. Ridge regression modeling, performance evaluation (weighted Pearson correlation), and feature importance ranking followed the procedures detailed in the *Drug response predictions* and *Feature selection and processing* sections, except that each model was run three times instead of ten.

### Cancer-type mapping analysis

To map candidate biomarkers to tumor lineages and nominate context-of-use, we evaluated associations between gene expression and drug response (AUC) within cancer types. Analyses focused on the top candidate genes highlighted in Fig 3D. Pearson and Spearman correlations were computed, with p-values adjusted using the Benjamini-Hochberg method. Heatmaps were generated for biomarker-drug pairs with ≥10 cell lines per cancer type, and a comprehensive table of results (including pairs with ≥3 cell lines) is provided in S7 Table.

### I-SPY2 clinical trial data

We obtained normalized and batch-corrected gene expression data from the I-SPY2 neoadjuvant breast cancer trial (NCT01042379) [67, 68] via the Gene Expression Omnibus (GEO, GSE194040). The analysis focused on patients in the paclitaxel treatment arm (n=179), which represents the control arm in the trial. We used pathologic complete response (pCR), the absence of residual invasive disease in both breast and lymph nodes, as the primary clinical endpoint. Clinical annotations, including treatment arm assignments and molecular subtypes, were retrieved from the accompanying metadata file (https://ars.els-cdn.com/content/image/1-s2.0-S1535610822002161-mmc3.xlsx). We classified patients with pCR = 1 as responders and those with pCR = 0 as non-responders. In line with our framework, we mapped paclitaxel to the mitosis-targeting drug class, for which *BID* expression emerged as a pan-cancer biomarker in the GDSC analysis (Fig 3D).

### Cell culture

A375 melanoma cells (source: ATCC) were cultured in Gibco Dulbecco’s Modified Eagle Medium supplemented with 10% Fetal Bovine Serum and 1% Penicillin-Streptomycin (10000 U/mL) in a humidified incubator (37°C, 5% CO2).

### siRNA mediated knockdown

10.000 A375 melanoma cells per well were reverse transfected in an opaque, white, flat-bottom plate, using *SLFN11* Silencer Select Pre-designed siRNA (Ambion: 4392420) and Silencer Negative Control siRNA #1 (Ambion: AM4611) with Lipofectamine RNAiMAX transfection reagent (Invitrogen: 13778075) and Gibco Opti-MEM reduced serum medium, following the manufacturer’s protocol for 1.5 pmol siRNA per well.

### Gel electrophoresis and western blotting

Cells were lysed using co-immunoprecipitation buffer (150mM NaCl, 25mM HEPES, 0.2% NP40, 1mM Glycerol) supplemented with cOmplete Protease Inhibitor Cocktail (Roche: 11836145001) and the protein concentrations were analyzed using Quick-Start Bradford 1X Dye Reagent (Bio-Rad: 5000205). The proteins were detected using anti-beta-Actin Antibody C4 (Santa Cruz: sc-47778) and anti-SLFN11 antibody (Abcam: ab121731).

### Drug treatment and dose response analysis

After the transfected cells were incubated overnight, they were treated with Gemcitabine (SelleckChem: S1714) dissolved in DMSO (0.5% v/v DMSO concentration per well). 72 hours after the treatment, cell viability was measured using CellTiter-Glo 2.0 Cell Viability Assay (Promega: G924A). Relative viability as a percentage of the negative control was calculated with intensities from blank (I_B_: medium only), negative control (I_NC_: DMSO treatment) and Gemcitabine treatment (I_G_) wells as:


$$Relative\;viability\;=\;\frac{I_{G}-\;I_{B}}{I_{NC}-\;I_{B}}$$

Dose-responses were analyzed using the four-parameter log-logistic (LL.4) model in the R package ‘drc’ [89].

## Supporting information

S1 Table Ridge, lasso and elasticNet gene expression model performance. (XLSX)

S2 Table Informative model (n = 266) built on gene expression, z-score-, and residual-corrected gene expression performances. (XLSX)

S3 Table Overview of screened cell line coverage across tissue types and drugs in the GDSC dataset. (XLSX)

S4 Table Delta Pearson and weighted Pearson correlation between baseline gene expression and z-score-corrected models across 385 drugs. (XLSX)

S5 Table Top 10 genes retained in both raw and z-score-corrected models, summarized by pathway and corresponding drugs. (XLSX)

S6 Table Gene-wise η² values representing the proportion of expression variance explained by tissue of origin across all genes. (XLSX)

S7 Table Cancer-type-specific correlations between candidate biomarker, SLFN11, ERBB2, IVL, BID, SPRY4, NES, and MAOB, expression and drug response, with adjusted p-values. (XLSX)

S1 Raw Images Original uncropped and unadjusted blot image corresponding to Figure 3, panel I. (PDF)

S1 Fig Principal Component Analysis plots and heatmap depicting gene expression data. (A) Gene expression data; (B) z-score corrected gene expression data; (C) residual corrected gene expression data (colored by cancer types); (D) residual corrected gene expression data (colored by cancer tumor types).
(PDF)

S2 Fig Method selection and model performance. (A) Performance of models built with ridge, lasso and (B) elasticNet regressions using tissue label data; (C) performance of models built with ridge, lasso and (D) elasticNet regressions using gene expression data; (E) Unweighted and (F) weighted Pearson correlation of models built with tissue labels, gene expression and residual corrected gene expression; observed and predicted AUC values with tissue labels models for (G) GSK2606414, (H) JQ1, (I) imatinib and (J) GNF-2.
(PDF)

S3 Fig Difference in drug response IC50s between non-solid and solid tumor types. (A) Mean difference between drug (n = 385) IC50s; density plots of IC50 of (B) tanespimycin, (C) bleomycin, (D) UNC0638, (E) vorinostat, (F) podophyllotoxin bromide, and (G) zoledronate.
(PDF)

S4 Fig Model selection. Distribution of weighted Pearson correlation of models built with (A) tissue labels alone (B) gene expression (C) residual corrected gene expression and (D) z-score corrected gene expression as well as respective null models (grey). (E) Overlap of informative models built using different modalities. (F) Overview of drug models which were classified as informative (n = 266) (not-informative in grey) stratified by pathway.
(PDF)

S5 Fig CTRP validation dataset. (A) Principal Component Analysis (PCA) plot depicting gene expression data coloured by the tissue origin of the cancer cell lines; (B) PCA plot depicting z-score corrected gene expression data; (C) an overlap of informative drug models with drugs screened in CTRP dataset; (D) number of drugs per pathway stratified by data source, GDSC (n = 266) and CTRP (n = 69).
(PDF)

S6 Fig NCI-60 dataset. (A) Principal Component Analysis (PCA) plot depicting gene expression data coloured by the tissue origin of the cancer cell lines; (B) PCA plot depicting z-score corrected gene expression data; ridge regression model performance after (C) 2, (D) 3, (E) 7, and (F) 11 days of drug exposure; average feature ranks of *SLFN11, BID, ERBB2, and IVL* across representative pathways for (G) day 2, (H) day 3, (I) day 7, and (J) day 11.
(PDF)

S7 Fig Robust pan-cancer biomarkers. (A) Percentage of drugs within pathway where specific gene is ranked in the first 10 positions; volcano plot of genes (n = 17) enriched in (B) GDSC and (C) CTRP drug pathways; rank of (D) *MAOB*, (E) *ERBB2*, (F) *BID* with DNA replication targeting drugs, (G) *BID* with mitosis targeting drugs, and (H) *IVL* in models built with gene expression and z-score corrected gene expression inputs. (I) Efficacy of Gemcitabine on *SLFN11* knockdown (blue) and negative control (grey) A375 melanoma cells. Wilcoxon test, ns: p > 0.05,*: p <= 0.05,**: p <= 0.01. (J) I-SPY2 paclitaxel arm (n = 179) validation of the association between *BID* expression and treatment response (responders, pCR = 1; non-responders, pCR = 0).
(PDF)

S8 Fig Assessment of feature stability and tissue attribution. (A) Mean proportion of retained and emerged top 10 gene features per drug pathway; (B) distribution of tissue attribution (η²) across all genes, and for retained and emerged top 10 features; (C) Δrank distribution for SLFN11, (D) SPRY4, (E) NES, and (F) ERBB2 showing rank improvement after tissue correction.
(PDF)

S9 Fig Gene-drug response associations for SLFN11. (A) Heatmap of Pearson r between gene expression and drug response (AUC) across cancer types (only drug-type pairs with n ≥ 10 are shown); scatterplots of SLFN11 expression and AUC for (B) gemcitabine (DNA replication) in GMB, and (C) camptothecin (DNA replication) in LIHC.
(PDF)

S10 Fig Gene-drug response associations for ERBB2. (A) Heatmap of Pearson r between gene expression and drug response (AUC) across cancer types (only drug-type pairs with n ≥ 10 are shown); scatterplots of ERBB2 expression and AUC for (B) osimertinib (EGFR signaling) in LUSC, and (C) afatinib (EGFR signaling) in BRCA.
(PDF)

S11 Fig Gene-drug response associations for SPRY4. (A) Heatmap of Pearson r between gene expression and drug response (AUC) across cancer types (only drug-type pairs with n ≥ 10 are shown); scatterplots of SPRY4 expression and AUC for (B) AZ628 (ERK MAPK signaling) in BRCA, and (C) ulixertinib (ERK MAPK signaling) in MM.
(PDF)

S12 Fig Gene-drug response associations for NES. (A) Heatmap of Pearson r between gene expression and drug response (AUC) across cancer types (only drug-type pairs with n ≥ 10 are shown); scatterplots of NES expression and AUC for (B) VX-11e (ERK MAPK signaling) in BLCA, and (C) PD0325901 (ERK MAPK signaling) in BLCA.
(PDF)

S13 Fig Gene-drug response associations for IVL. (A) Heatmap of Pearson r between gene expression and drug response (AUC) across cancer types (only drug-type pairs with n ≥ 10 are shown); scatterplots of IVL expression and AUC for (B) gefitinib (EGFR signaling) in CESC, and (C) AZD3759 (EGFR signaling) in CESC.
(PDF)

S14 Fig Gene-drug response associations for MAOB. (A) Heatmap of Pearson r between gene expression and drug response (AUC) across cancer types (only drug-type pairs with n ≥ 10 are shown); scatterplots of MAOB expression and AUC for (B) AZ628 (ERK MAPK signaling) in BRCA, and (C) dabrafenib (ERK MAPK signaling) in BRCA.
(PDF)

S15 Fig Gene-drug response associations for BID. (A) Heatmap of Pearson r between gene expression and drug response (AUC) across cancer types (only drug-type pairs with n ≥ 10 are shown); scatterplots of BID expression and AUC for (B) epothilone B (mitosis) in LGG, (C) docetaxel (mitosis) in SCLC, (D) bleomycin (DNA replication) in LGG, and (E) gemcitabine (DNA replication) in LGG.
(PDF)
