# Supplementary material for: Systematic identification of pan-cancer single-gene expression biomarkers in drug high-throughput screens
Source: PLoS One. 2026 May 11;21(5):e0330412. doi: 10.1371/journal.pone.0330412 (PMC13160354; doi:10.1371/journal.pone.0330412)
Supplement: S1 Raw Images — (PDF) [file pone.0330412.s008.pdf]

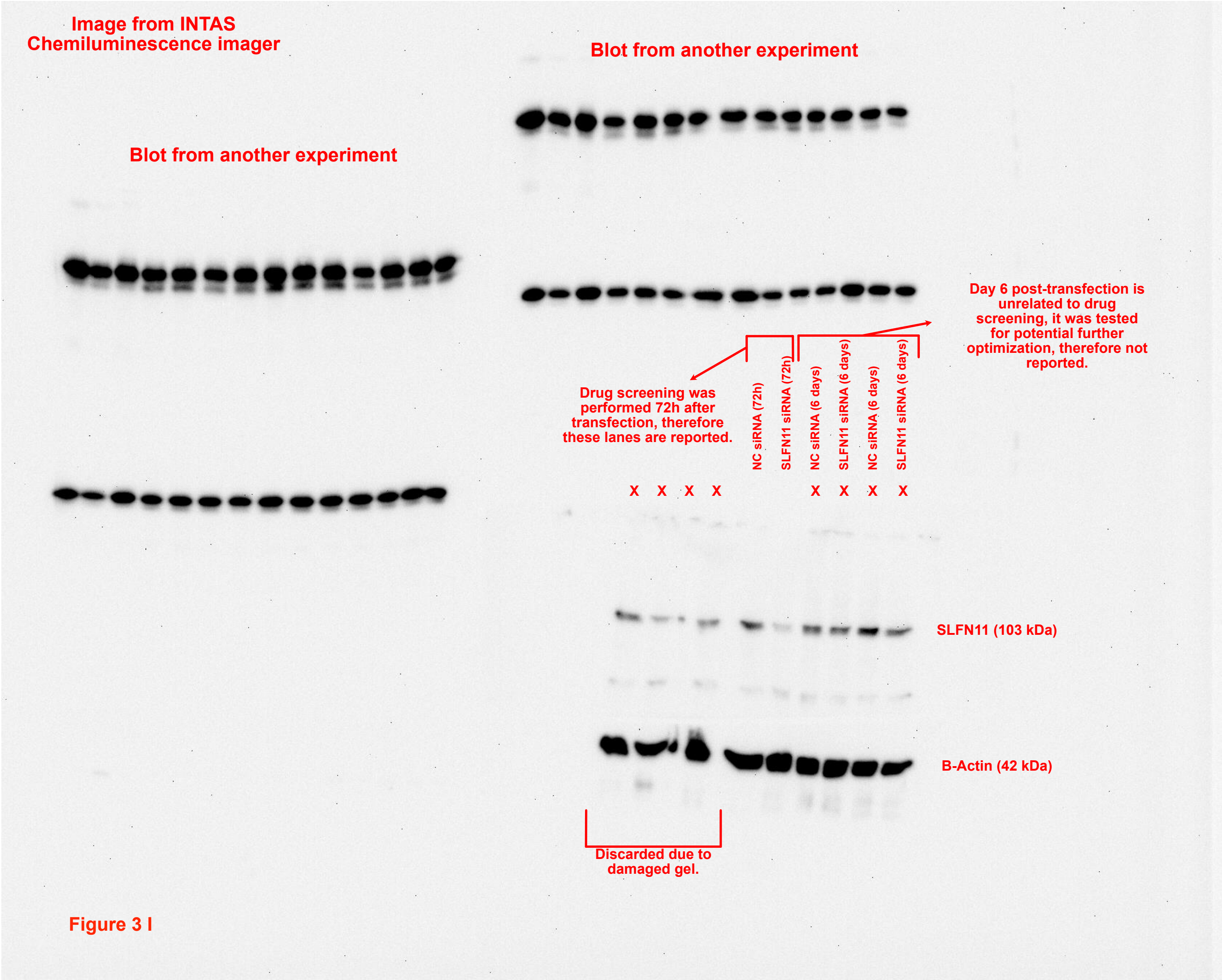
Image from INTAS
Chemiluminescence imager
Blot from another experiment
Blot from another experiment
Day 6 post-transfection is unrelated to drug screening, it was tested for potential further optimization, therefore not reported.
Drug screening was performed 72h after transfection, therefore these lanes are reported.
NC siRNA (72h)
SLFN11 siRNA (72h)
NC siRNA (6 days)
SLFN11 siRNA (6 days)
NC siRNA (6 days)
SLFN11 siRNA (6 days)
X X X X
X X X X
SLFN11 (103 kDa)
B-Actin (42 kDa)
Discarded due to damaged gel.
Figure 3 I
