# Supplementary material for: Systematic identification of pan-cancer single-gene expression biomarkers in drug high-throughput screens
Source: PLoS One. 2026 May 11;21(5):e0330412. doi: 10.1371/journal.pone.0330412 (PMC13160354; doi:10.1371/journal.pone.0330412)
Supplement: S1 Fig — (A) Gene expression data; (B) z-score corrected gene expression data; (C) residual corrected gene expression data (colored by cancer types); (D) residual corrected gene expression data (colored by cancer tumor types). (PDF) [file pone.0330412.s009.pdf]

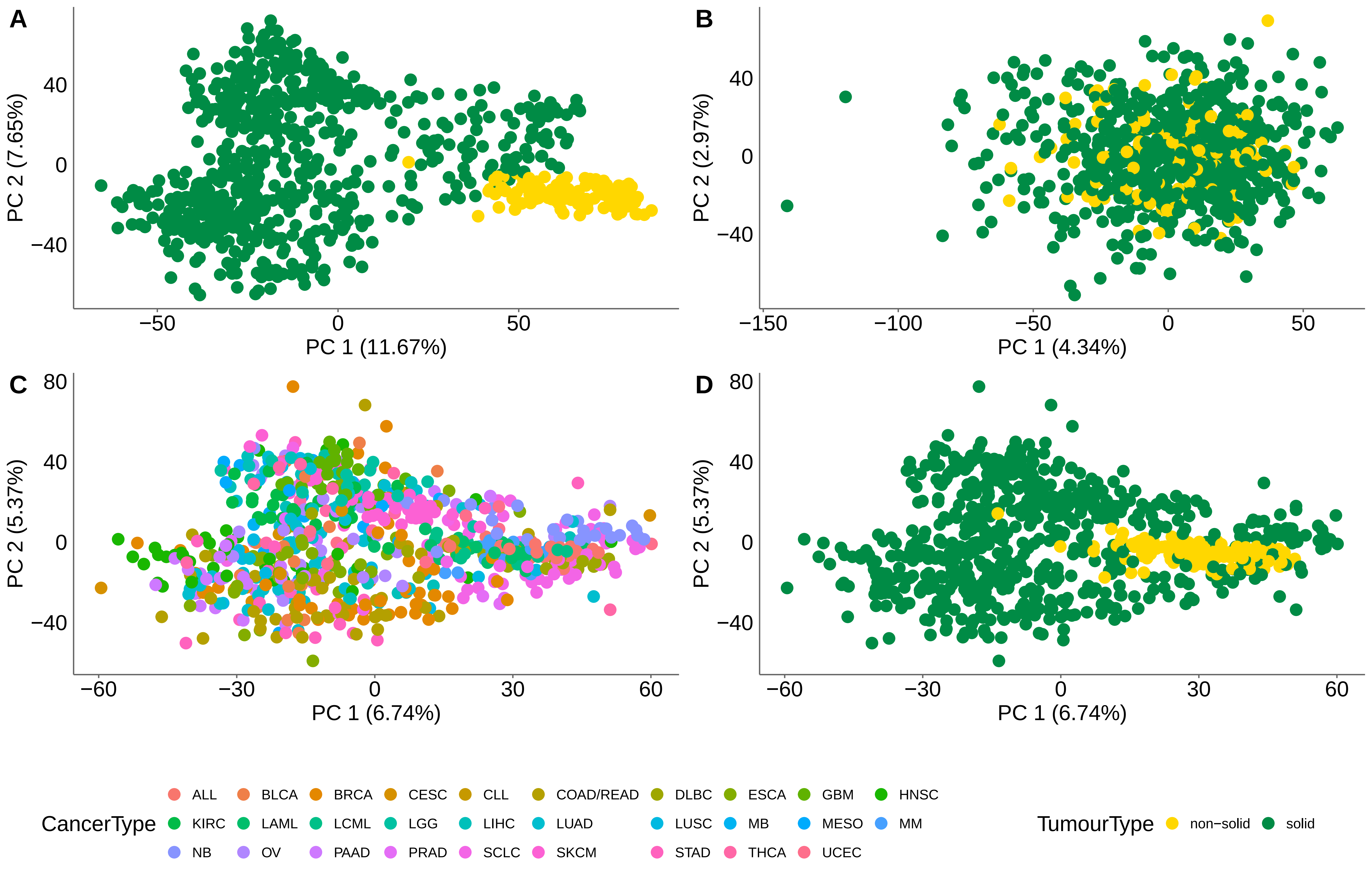

A
B
C
D
PC 1 (11.67%)
PC 2 (7.65%)
PC 1 (4.34%)
PC 2 (2.97%)
PC 1 (6.74%)
PC 2 (5.37%)
PC 1 (6.74%)
PC 2 (5.37%)
CancerType
ALL
BLCA
BRCA
CESC
CLL
COAD/READ
DLBC
ESCA
GBM
HNSC
KIRC
LAML
LCML
LGG
LIHC
LUAD
LUSC
MB
MESO
MM
NB
OV
PAAD
PRAD
SCLC
SKCM
STAD
THCA
UCEC
TumourType
non−solid
solid
