# Supplementary material for: Systematic identification of pan-cancer single-gene expression biomarkers in drug high-throughput screens
Source: PLoS One. 2026 May 11;21(5):e0330412. doi: 10.1371/journal.pone.0330412 (PMC13160354; doi:10.1371/journal.pone.0330412)
Supplement: S2 Fig — (A) Performance of models built with ridge, lasso and (B) elasticNet regressions using tissue label data; (C) performance of models built with ridge, lasso and (D) elasticNet regressions using gene expression data; (E) Unweighted and (F) weighted Pearson correlation of models built with tissue labels, gene expression and residual corrected gene expression; observed and predicted AUC values with tissue labels models for (G) GSK2606414, (H) JQ1, (I) imatinib and (J) GNF-2. (PDF) [file pone.0330412.s010.pdf]

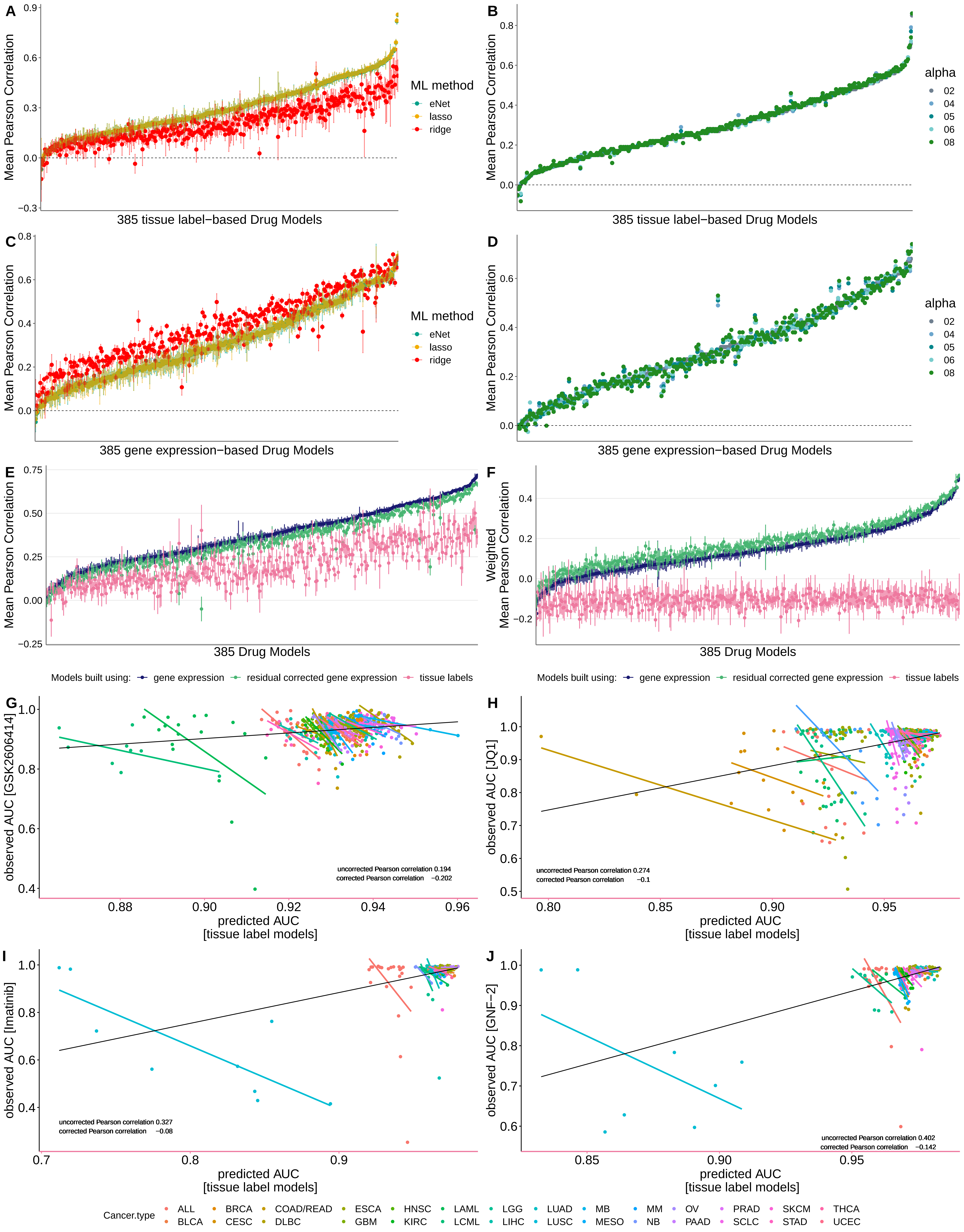

A
Mean Pearson Correlation
385 tissue label−based Drug Models
ML method
eNet
lasso
ridge
B
Mean Pearson Correlation
385 tissue label−based Drug Models
alpha
02
04
05
06
08
C
Mean Pearson Correlation
385 gene expression−based Drug Models
ML method
eNet
lasso
ridge
D
Mean Pearson Correlation
385 gene expression−based Drug Models
alpha
02
04
05
06
08
E
Mean Pearson Correlation
385 Drug Models
F
Weighted Mean Pearson Correlation
385 Drug Models
Models built using: gene expression residual corrected gene expression tissue labels
Models built using: gene expression residual corrected gene expression tissue labels
G
observed AUC [GSK2606414]
uncorrected Pearson correlation 0.194
corrected Pearson correlation −0.202
predicted AUC [tissue label models]
H
observed AUC [JQ1]
uncorrected Pearson correlation 0.274
corrected Pearson correlation −0.1
predicted AUC [tissue label models]
I
observed AUC [Imatinib]
uncorrected Pearson correlation 0.327
corrected Pearson correlation −0.08
predicted AUC [tissue label models]
J
observed AUC [GNF−2]
uncorrected Pearson correlation 0.402
corrected Pearson correlation −0.142
predicted AUC [tissue label models]
Cancer.type
ALL
BLCA
BRCA
CESC
COAD/READ
DLBC
ESCA
GBM
HNSC
KIRC
LAML
LCML
LGG
LIHC
LUAD
LUSC
MB
MESO
MM
NB
OV
PAAD
PRAD
SCLC
SKCM
STAD
THCA
UCEC
