# Supplementary figures and images for: Systematic identification of pan-cancer single-gene expression biomarkers in drug high-throughput screens
Source: PLoS One. 2026 May 11;21(5):e0330412. doi: 10.1371/journal.pone.0330412 (PMC13160354; doi:10.1371/journal.pone.0330412)

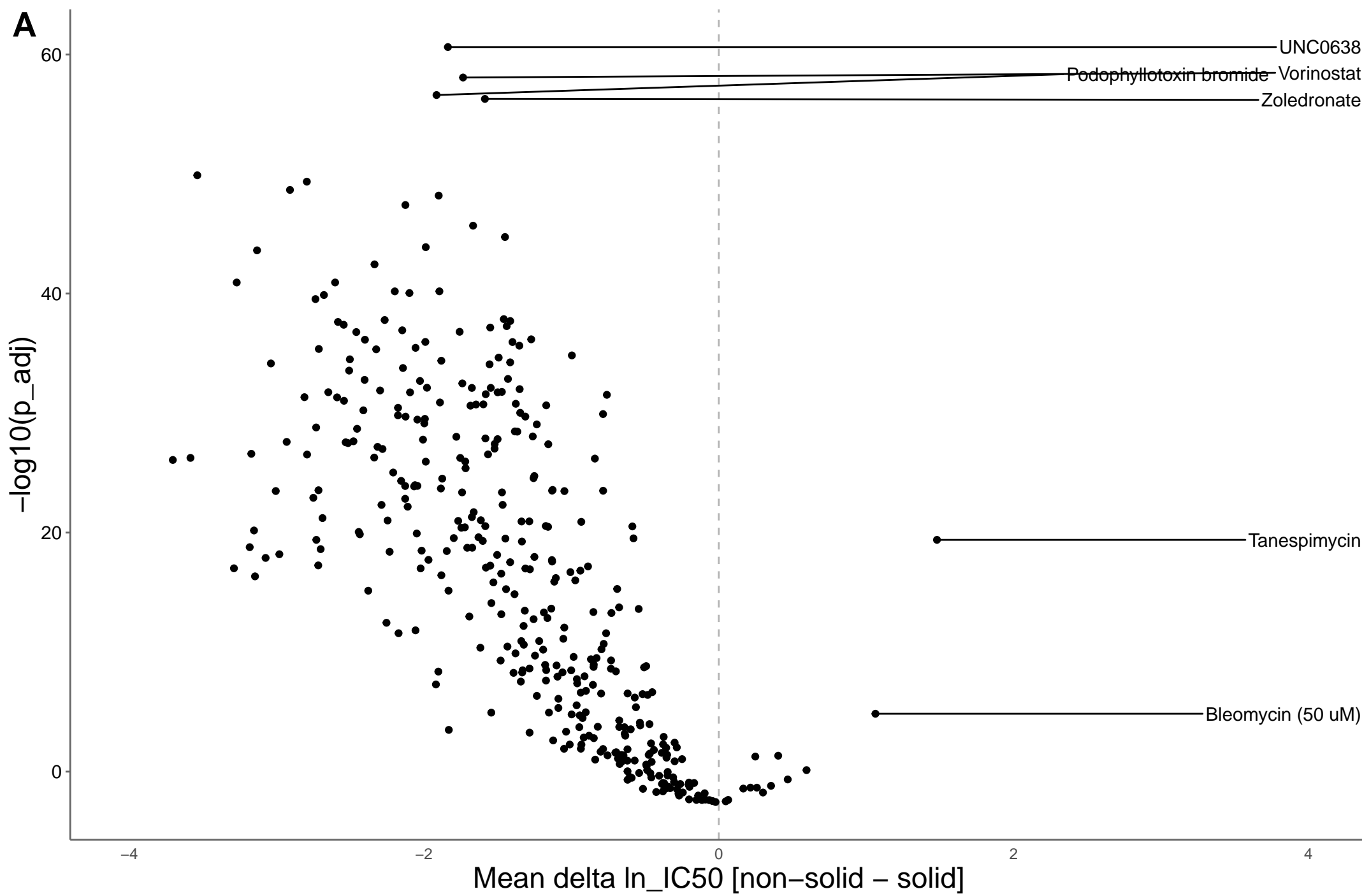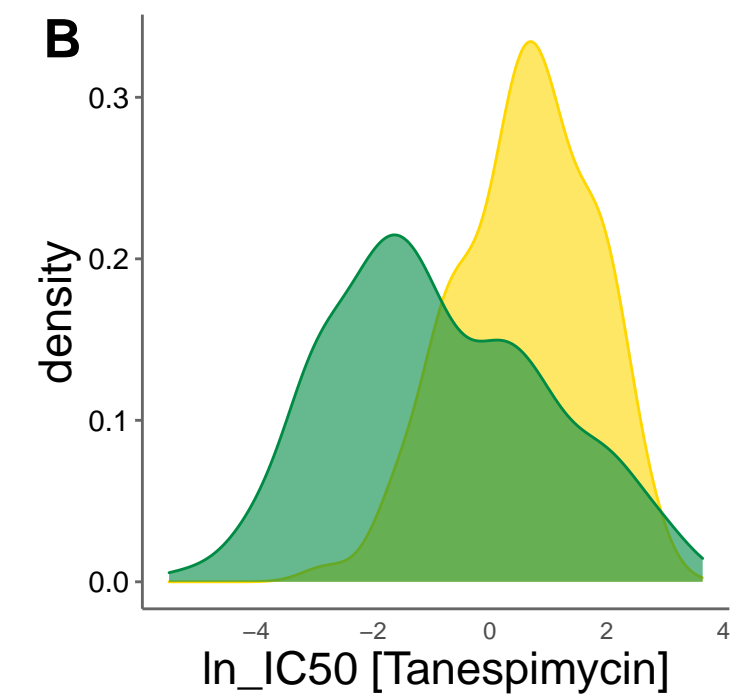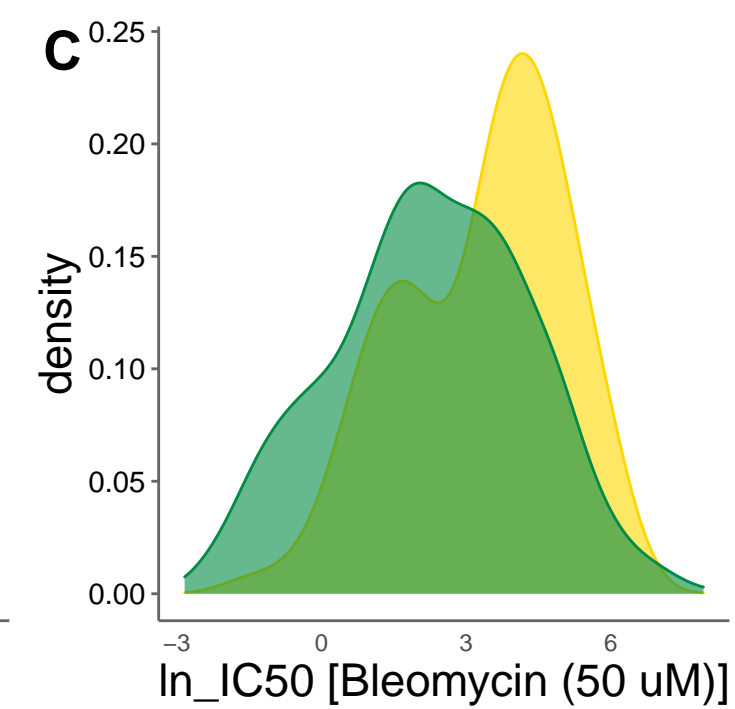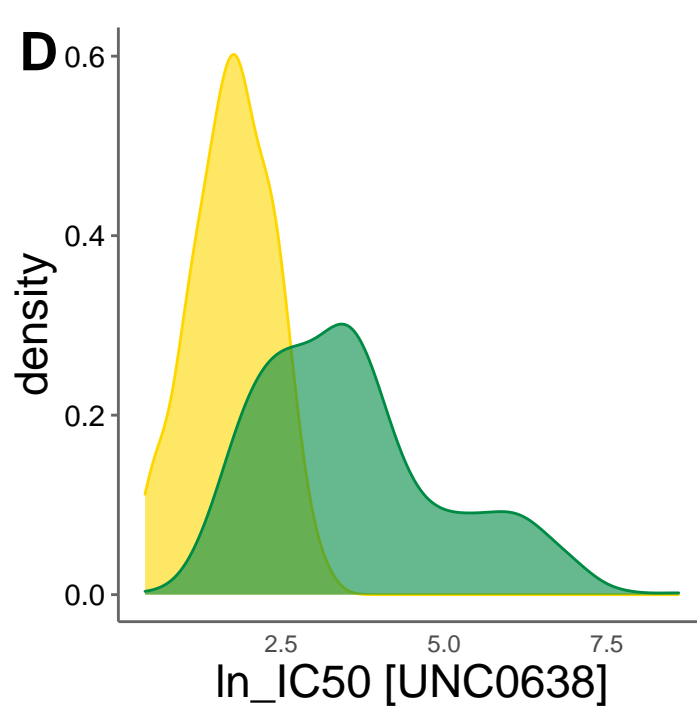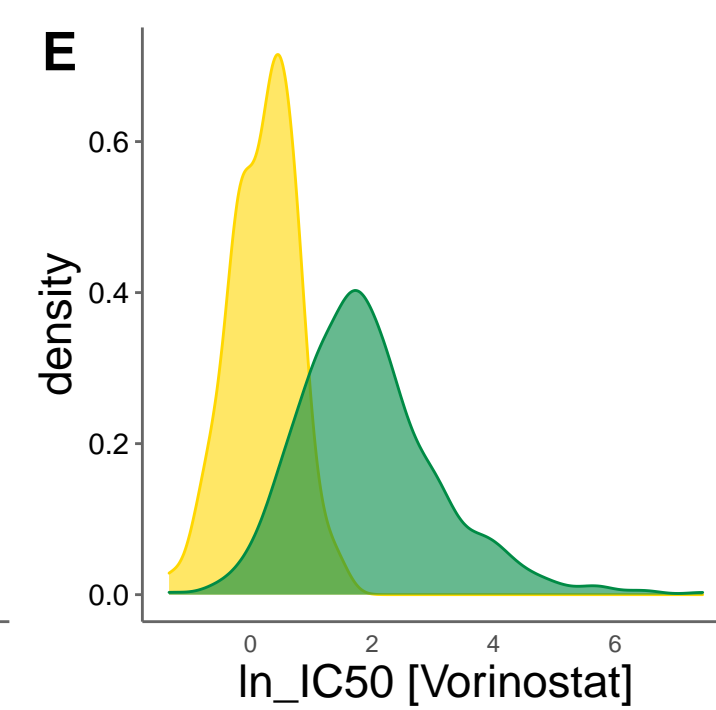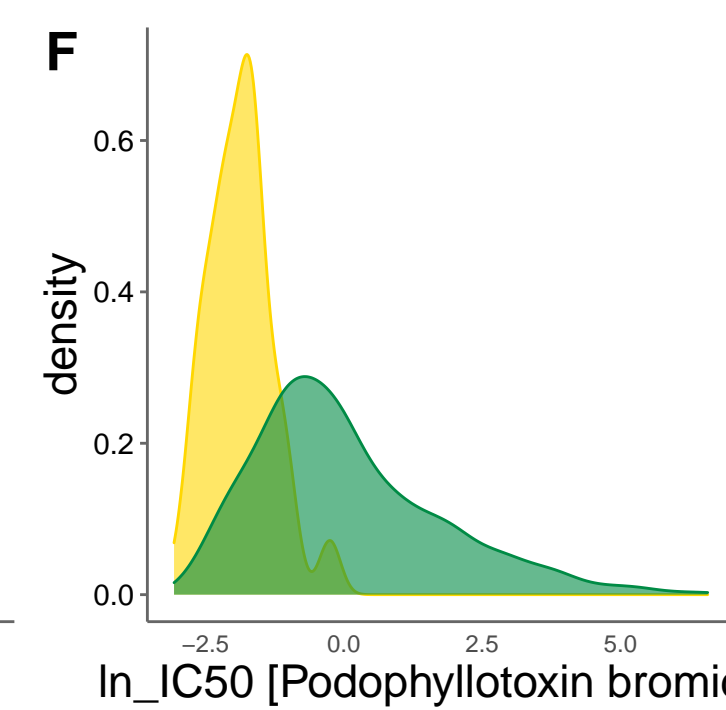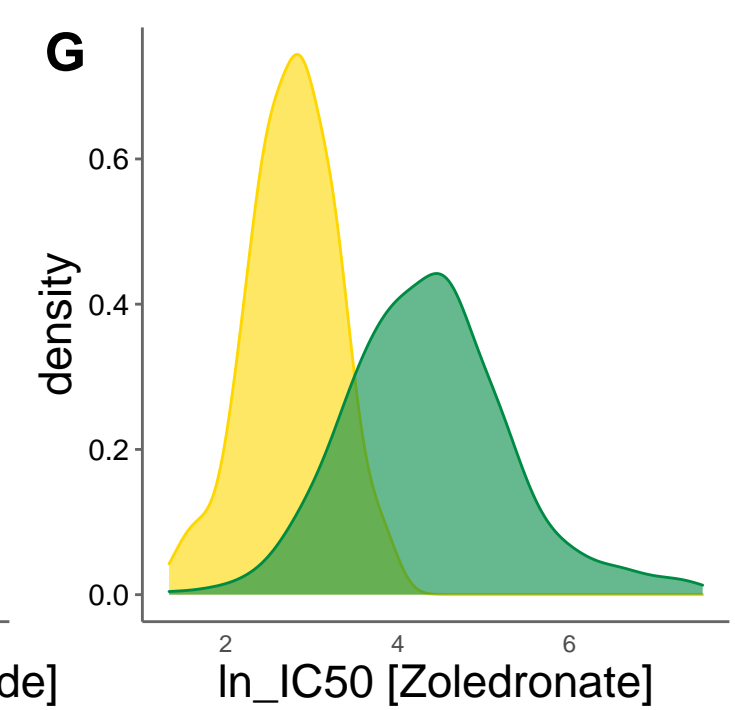

TumourType ■ non-solid ■ solid

Supplement: S3 Fig — (A) Mean difference between drug (n = 385) IC50s; density plots of IC50 of (B) tanespimycin, (C) bleomycin, (D) UNC0638, (E) vorinostat, (F) podophyllotoxin bromide, and (G) zoledronate. (PDF) [file pone.0330412.s011.pdf]

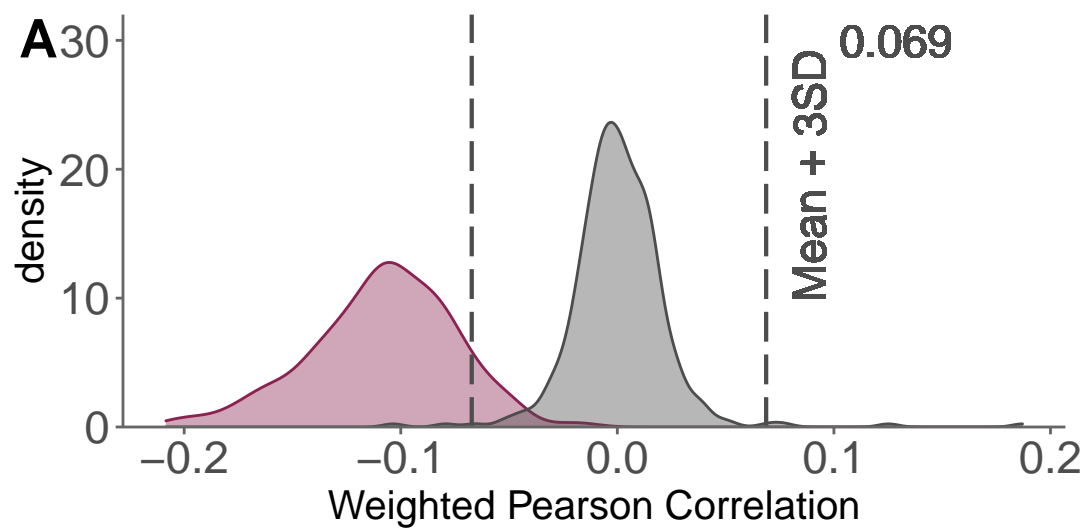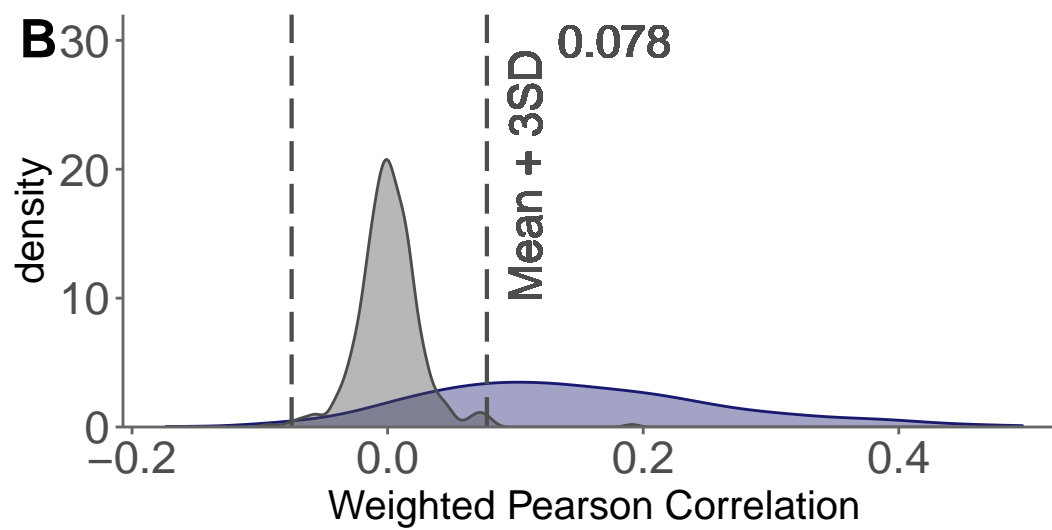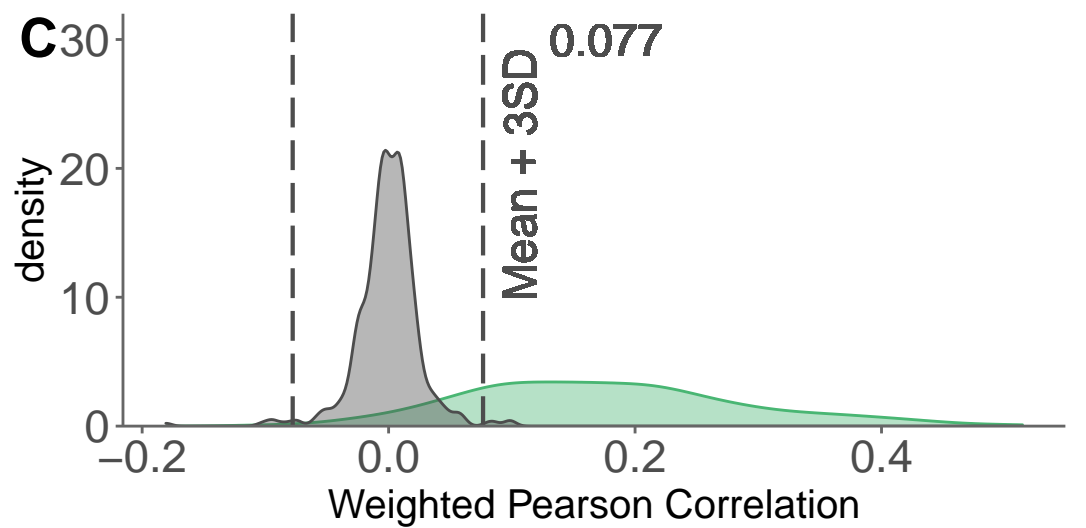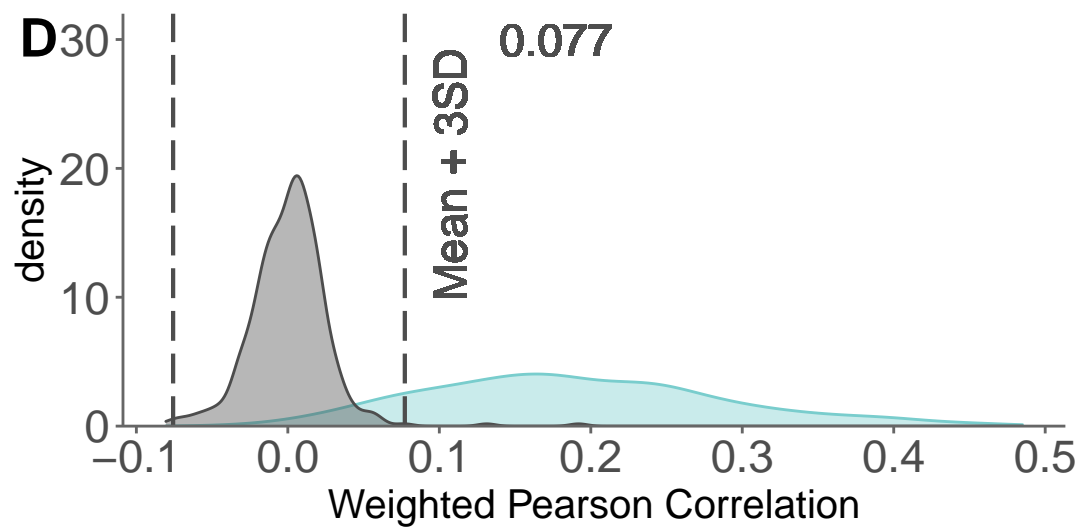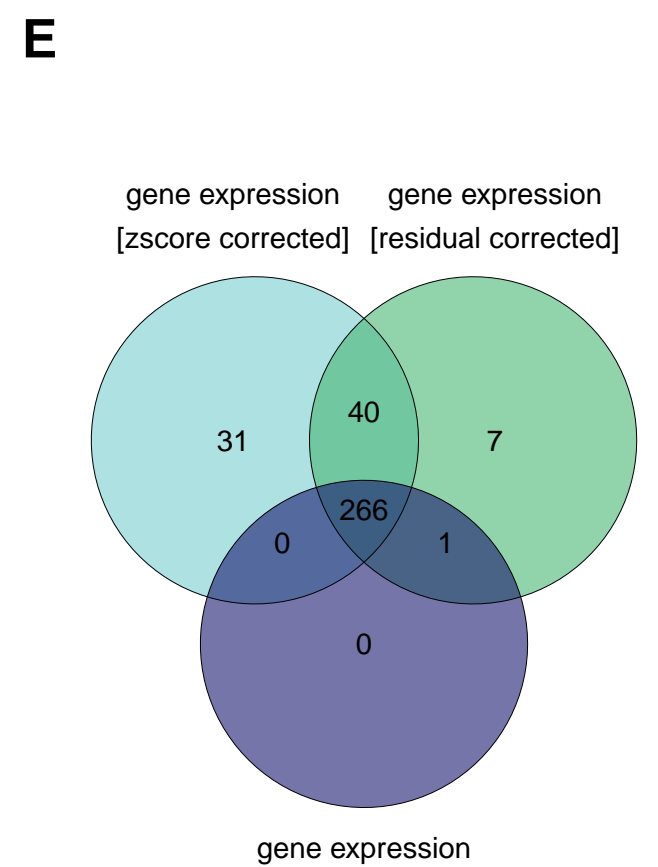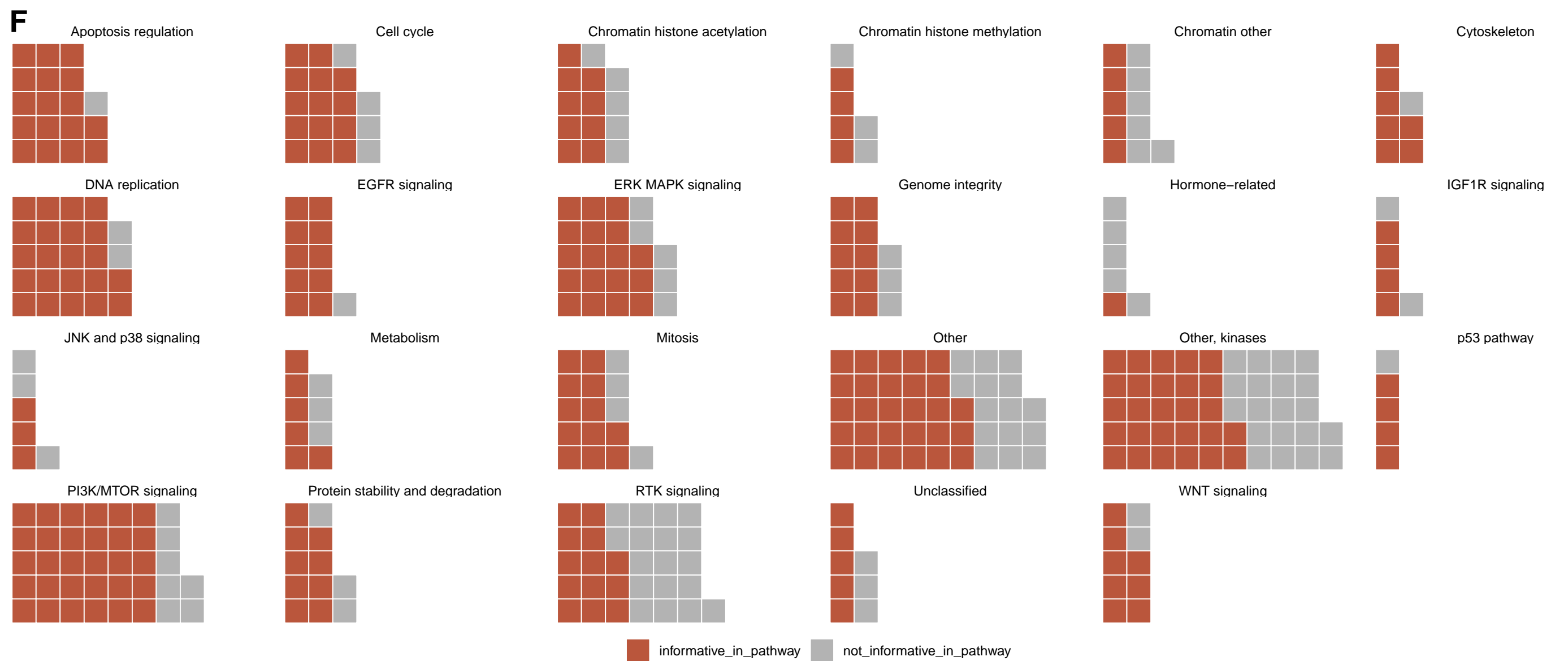

Supplement: S4 Fig — Distribution of weighted Pearson correlation of models built with (A) tissue labels alone (B) gene expression (C) residual corrected gene expression and (D) z-score corrected gene expression as well as respective null models (grey). (E) Overlap of informative models built using different modalities. (F) Overview of drug models which were classified as informative (n = 266) (not-informative in grey) stratified by pathway. (PDF) [file pone.0330412.s012.pdf]

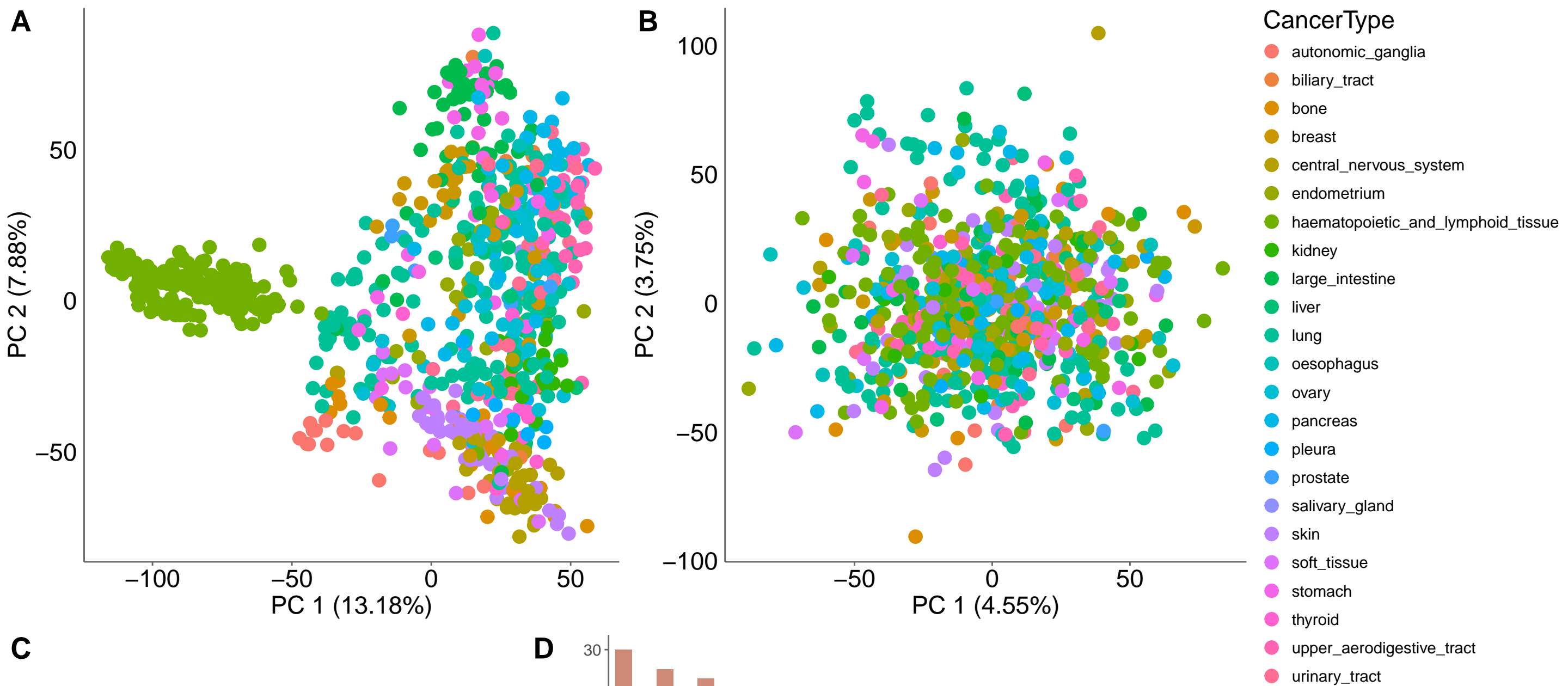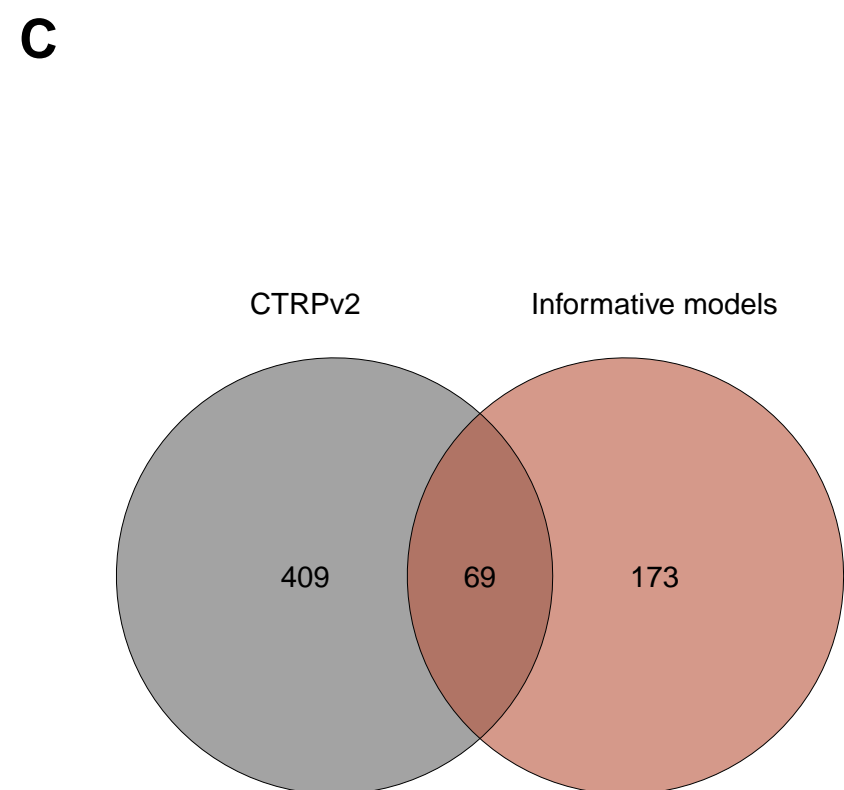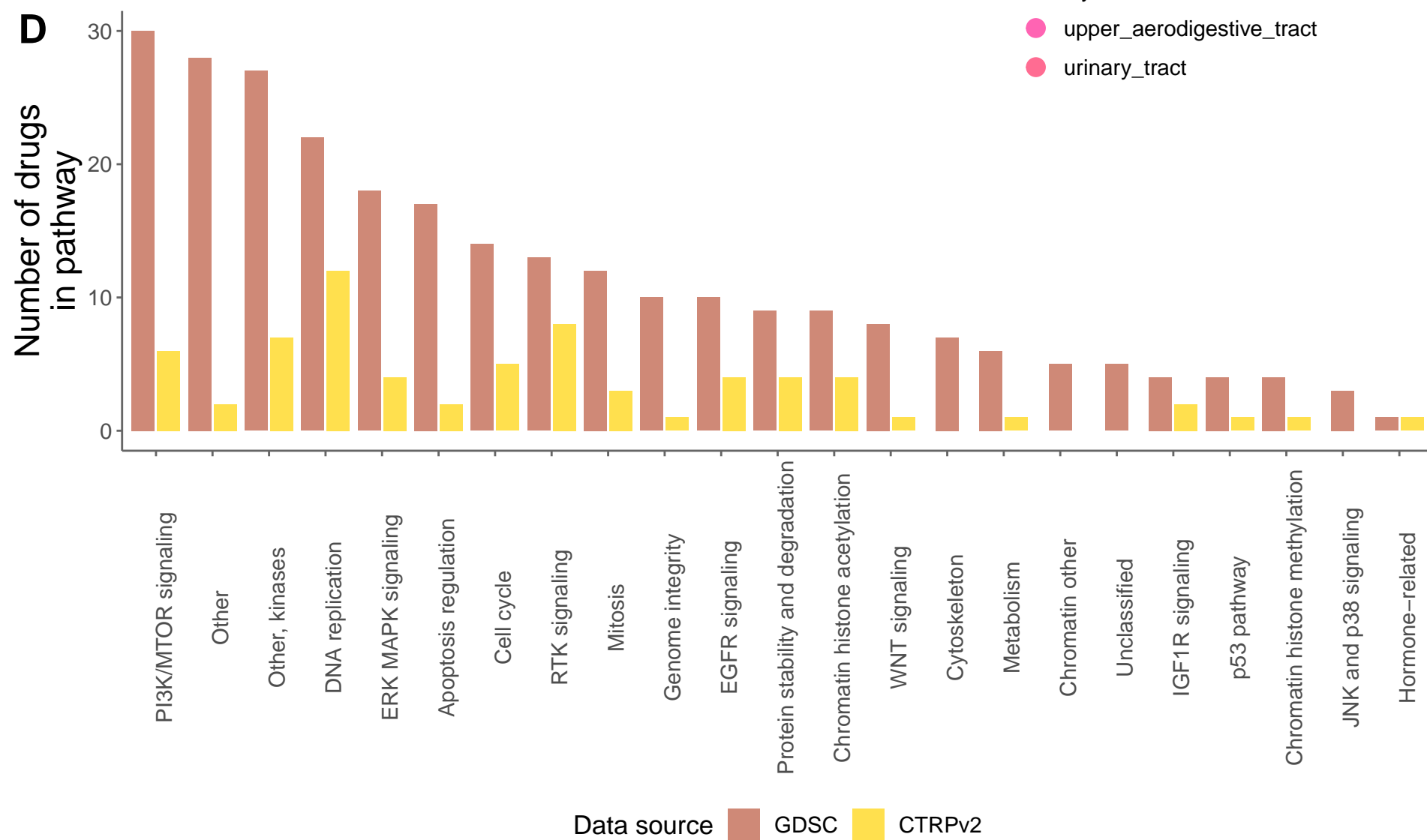

Supplement: S5 Fig — (A) Principal Component Analysis (PCA) plot depicting gene expression data coloured by the tissue origin of the cancer cell lines; (B) PCA plot depicting z-score corrected gene expression data; (C) an overlap of informative drug models with drugs screened in CTRP dataset; (D) number of drugs per pathway stratified by data source, GDSC (n = 266) and CTRP (n = 69). (PDF) [file pone.0330412.s013.pdf]

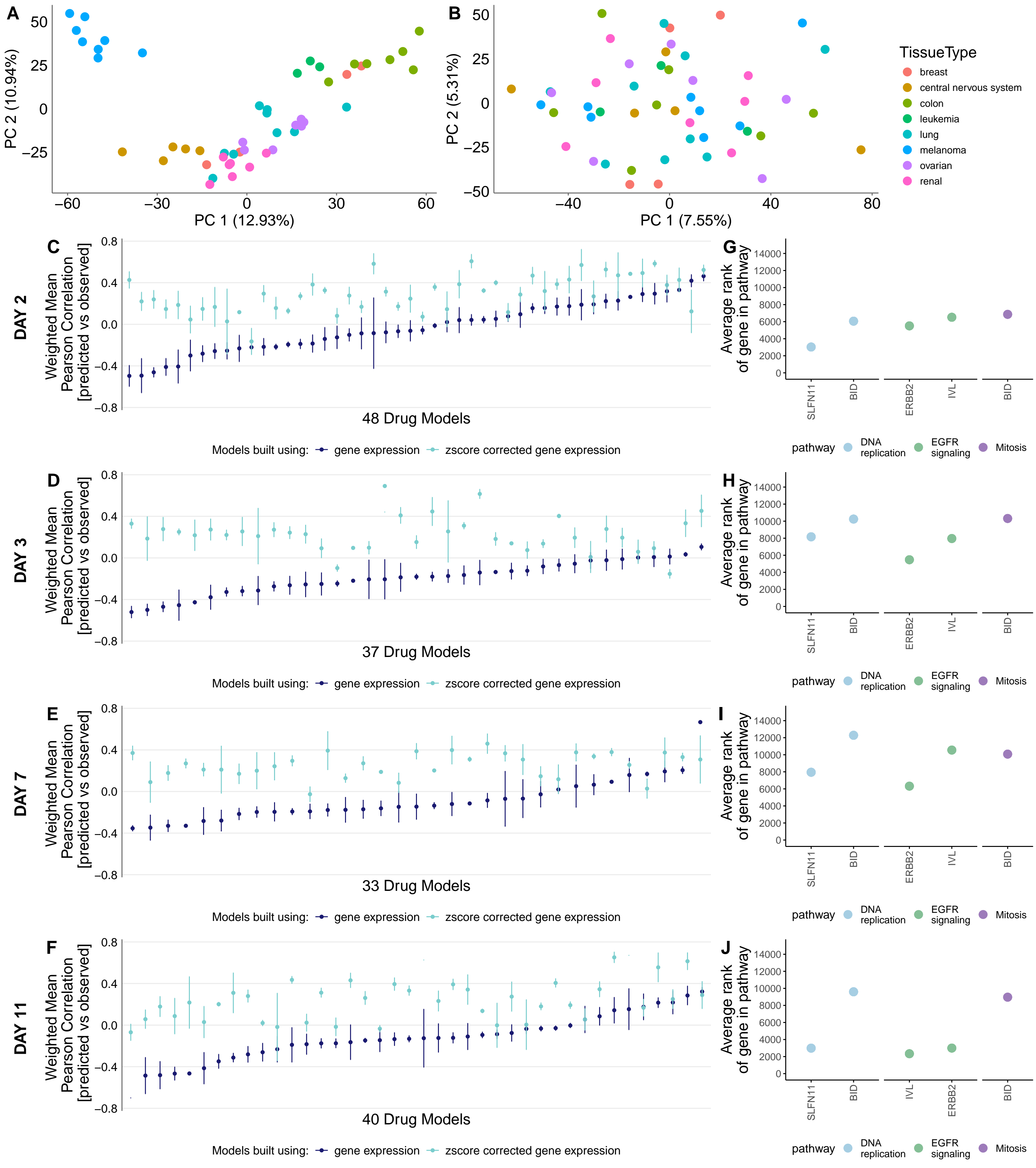

Supplement: S6 Fig — (A) Principal Component Analysis (PCA) plot depicting gene expression data coloured by the tissue origin of the cancer cell lines; (B) PCA plot depicting z-score corrected gene expression data; ridge regression model performance after (C) 2, (D) 3, (E) 7, and (F) 11 days of drug exposure; average feature ranks of SLFN11, BID, ERBB2, and IVL across representative pathways for (G) day 2, (H) day 3, (I) day 7, and (J) day 11. (PDF) [file pone.0330412.s014.pdf]

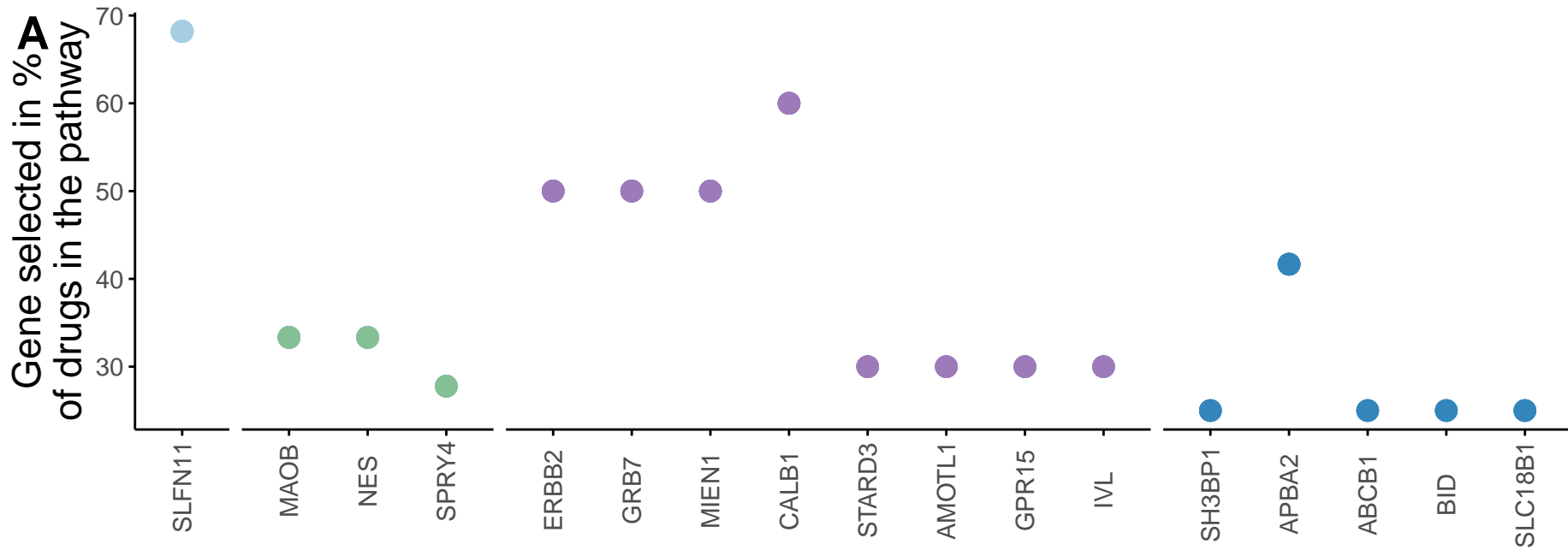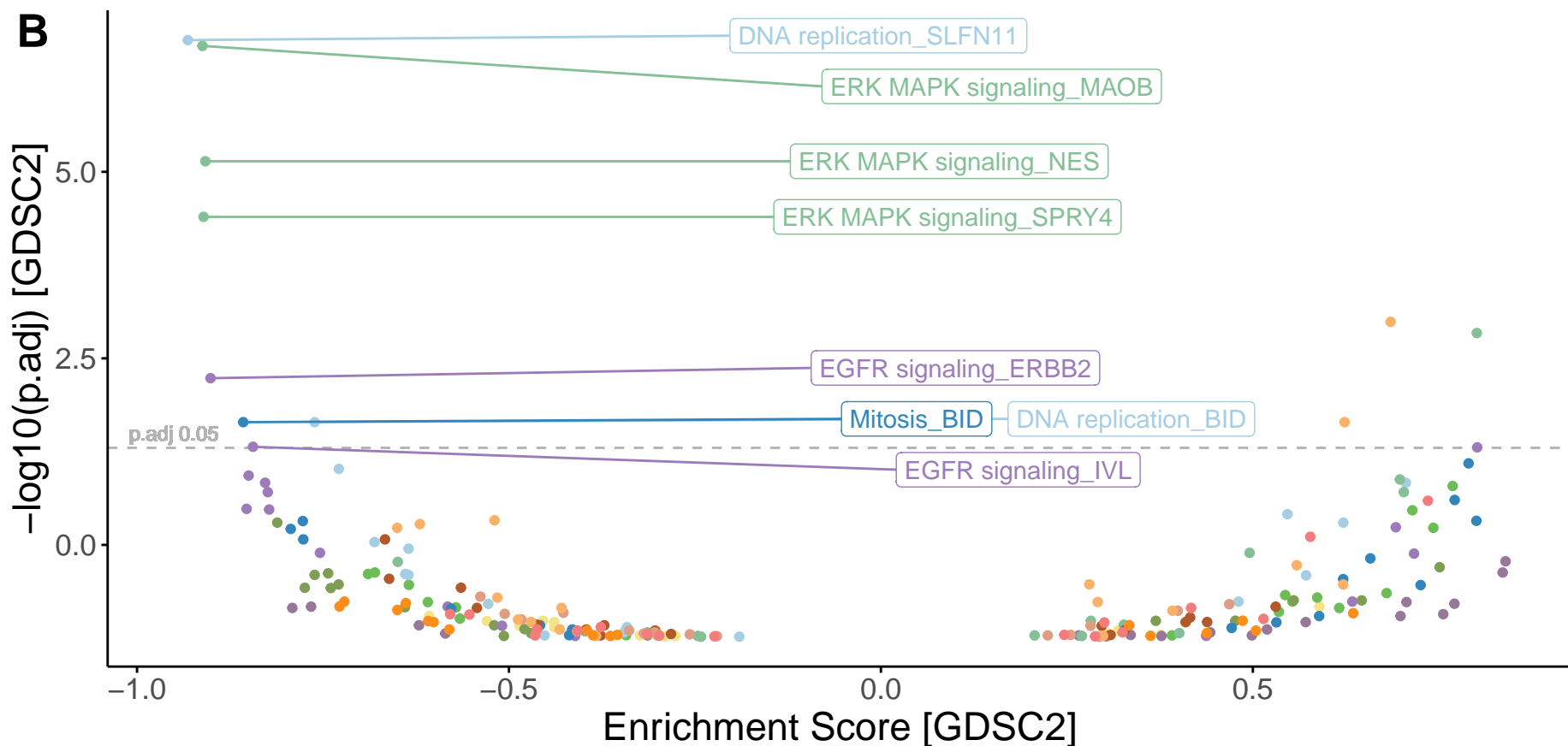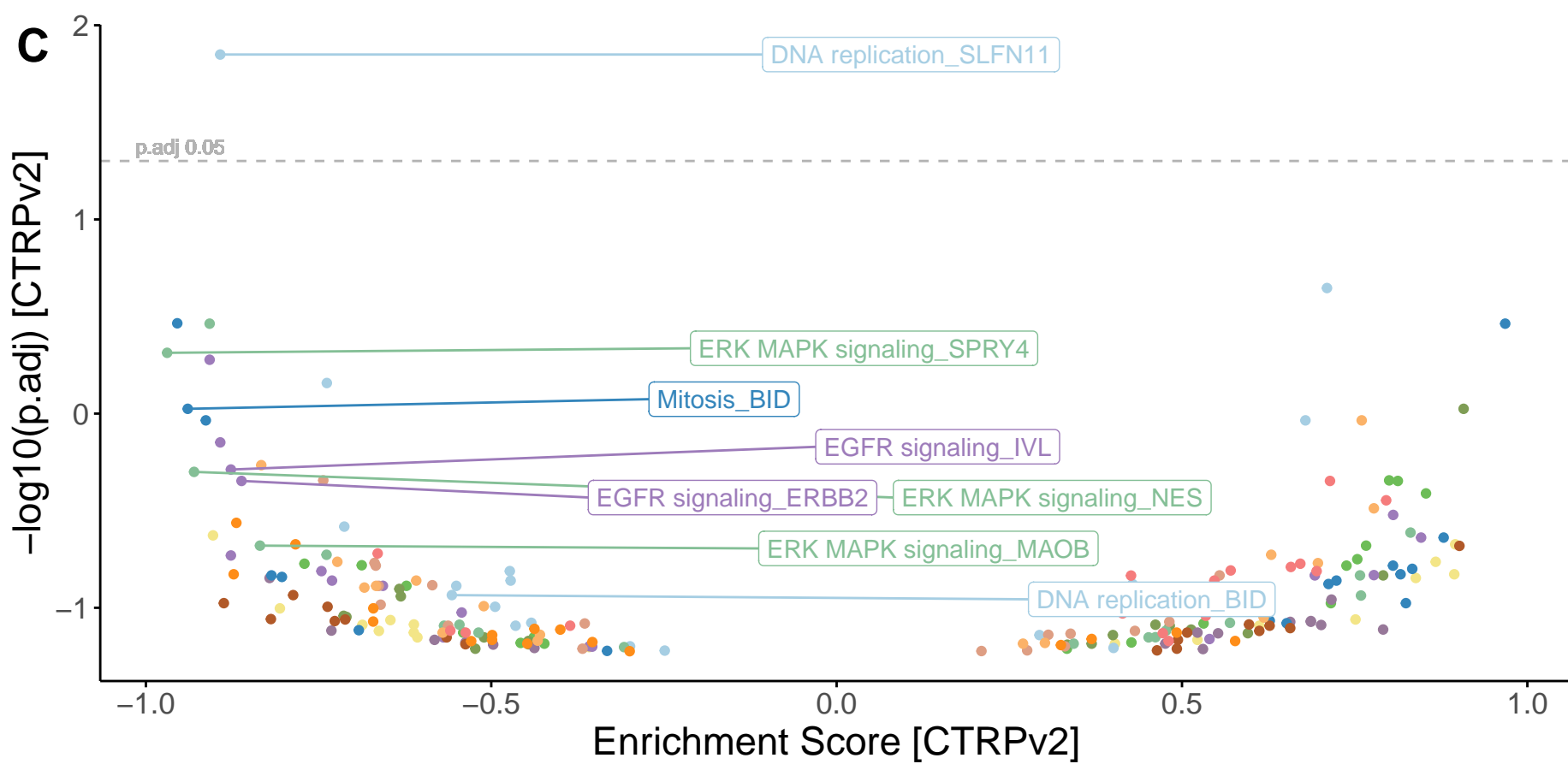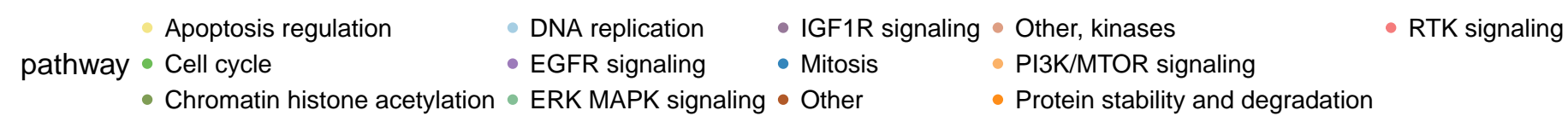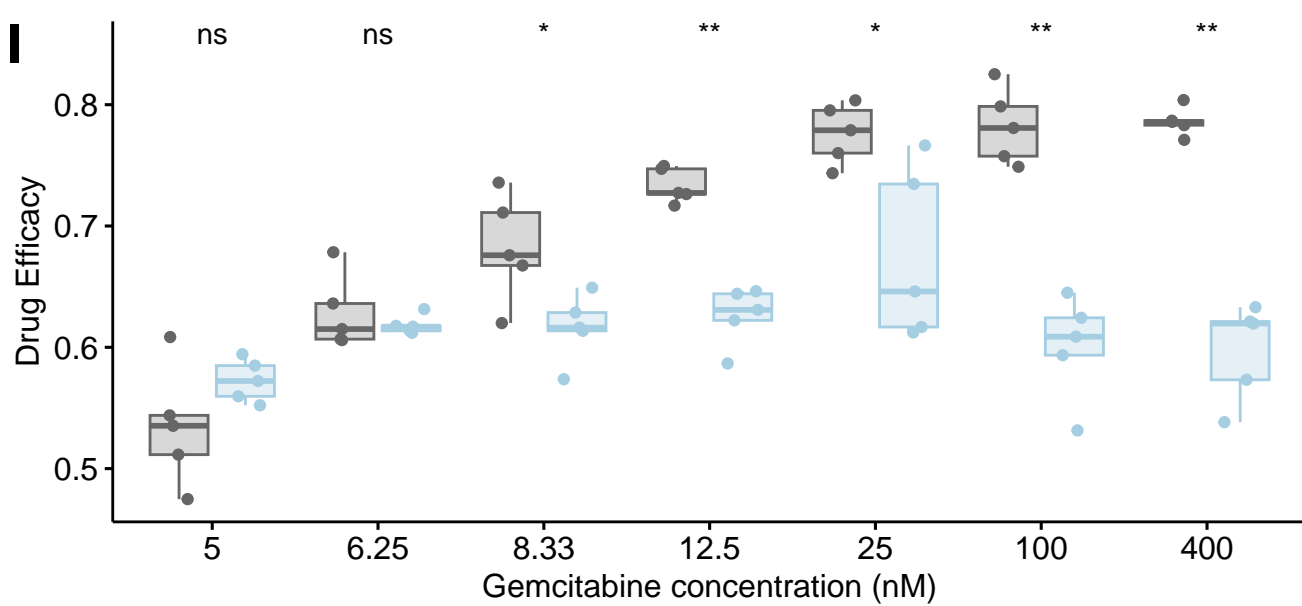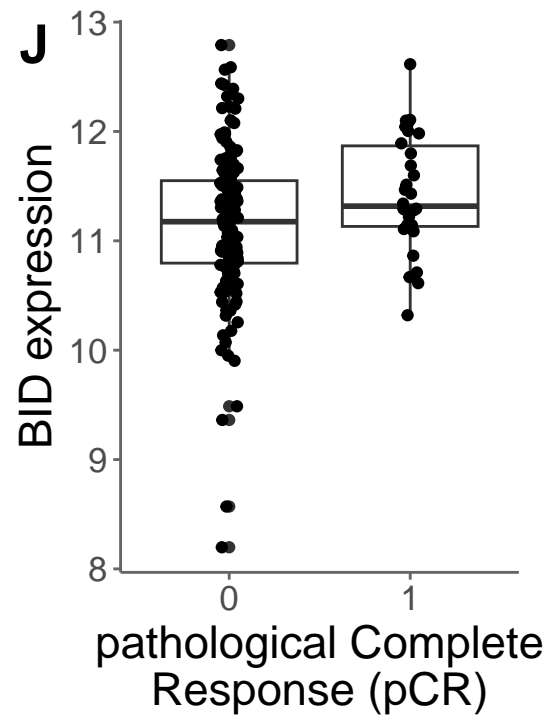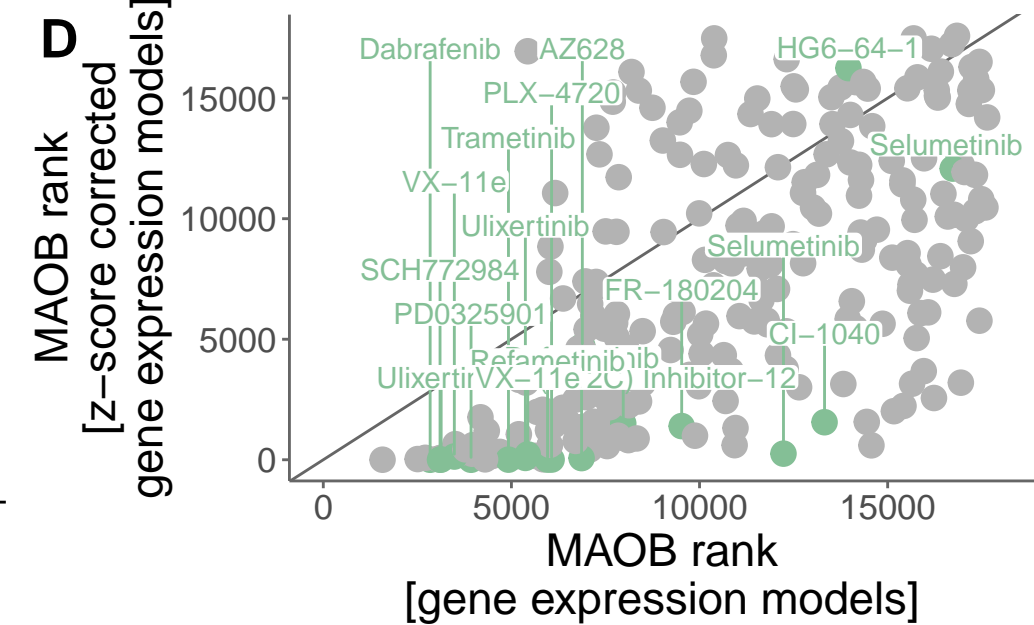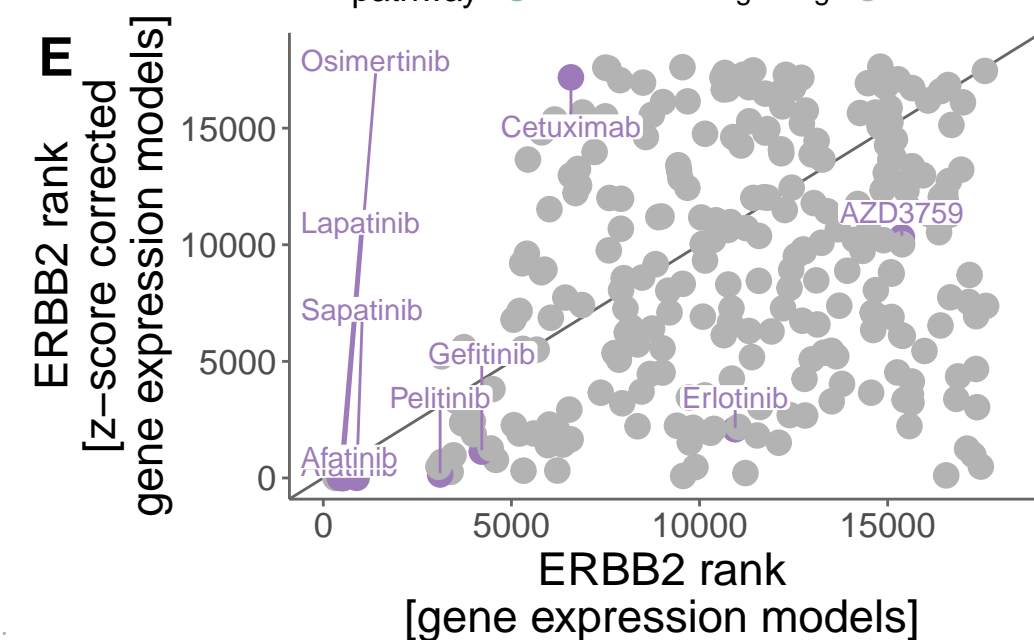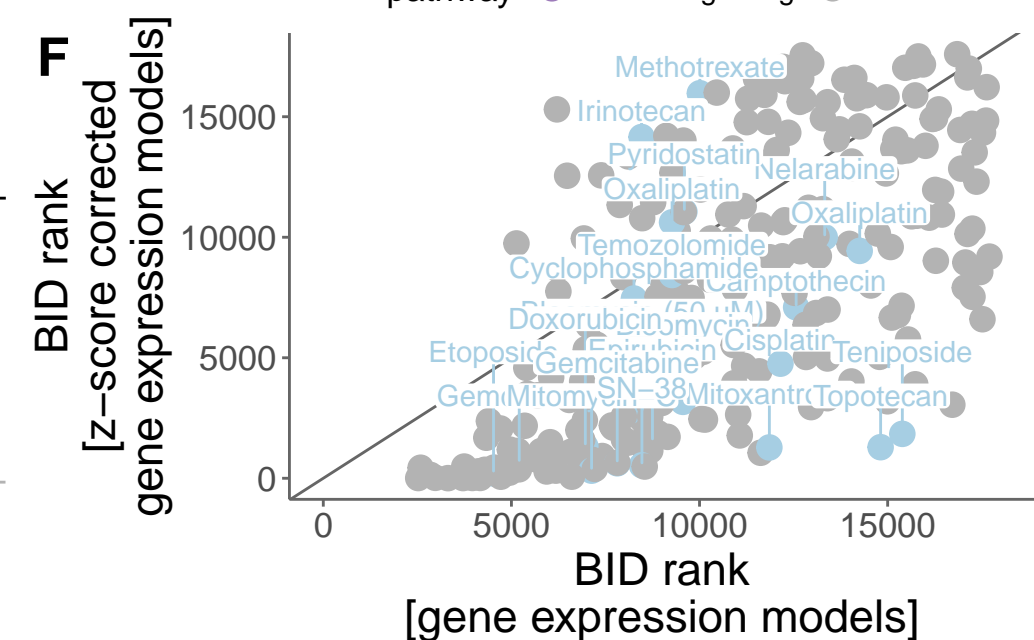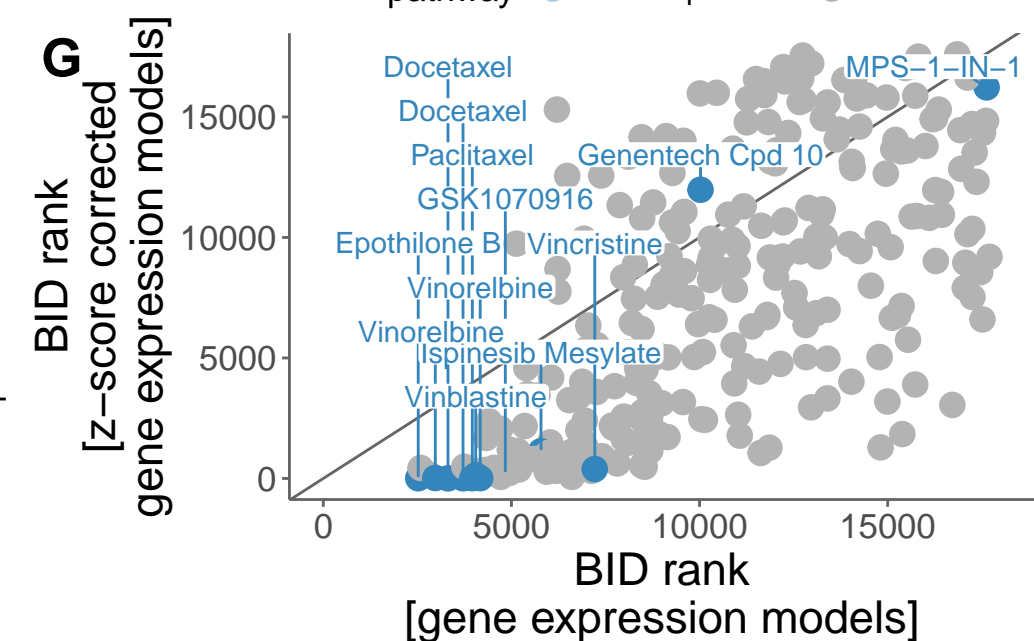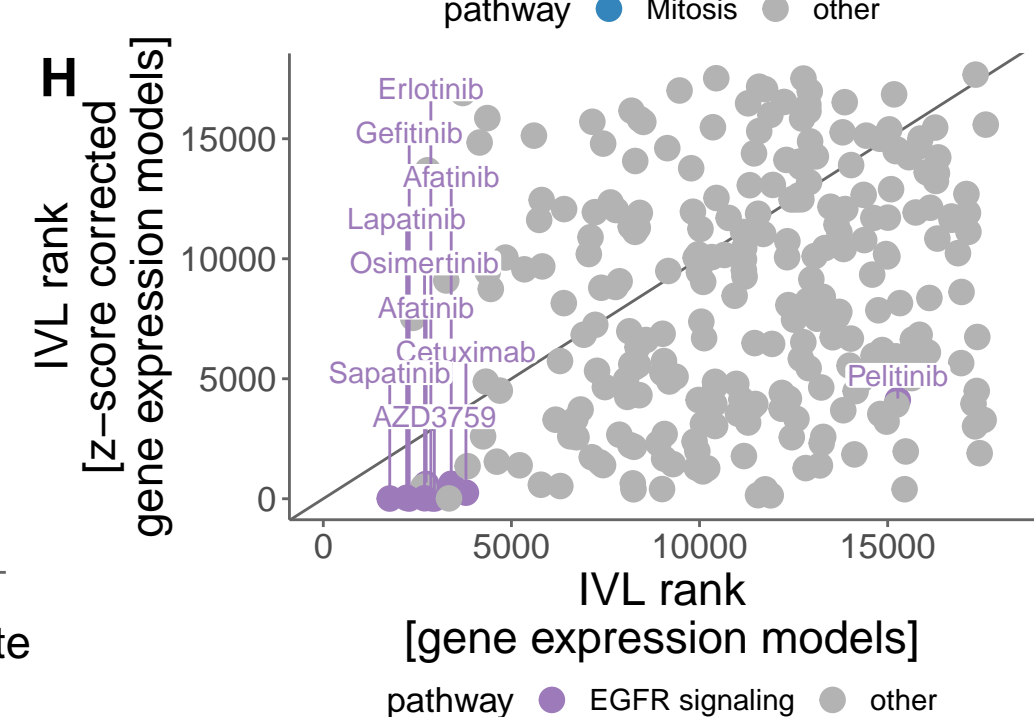

Supplement: S7 Fig — (A) Percentage of drugs within pathway where specific gene is ranked in the first 10 positions; volcano plot of genes (n = 17) enriched in (B) GDSC and (C) CTRP drug pathways; rank of (D) MAOB, (E) ERBB2, (F) BID with DNA replication targeting drugs, (G) BID with mitosis targeting drugs, and (H) IVL in models built with gene expression and z-score corrected gene expression inputs. (I) Efficacy of Gemcitabine on SLFN11 knockdown (blue) and negative control (grey) A375 melanoma cells. Wilcoxon test, ns: p > 0.05,*: p <= 0.05,**: p <= 0.01. (J) I-SPY2 paclitaxel arm (n = 179) validation of the association between BID expression and treatment response (responders, pCR = 1; non-responders, pCR = 0). (PDF) [file pone.0330412.s015.pdf]

**A**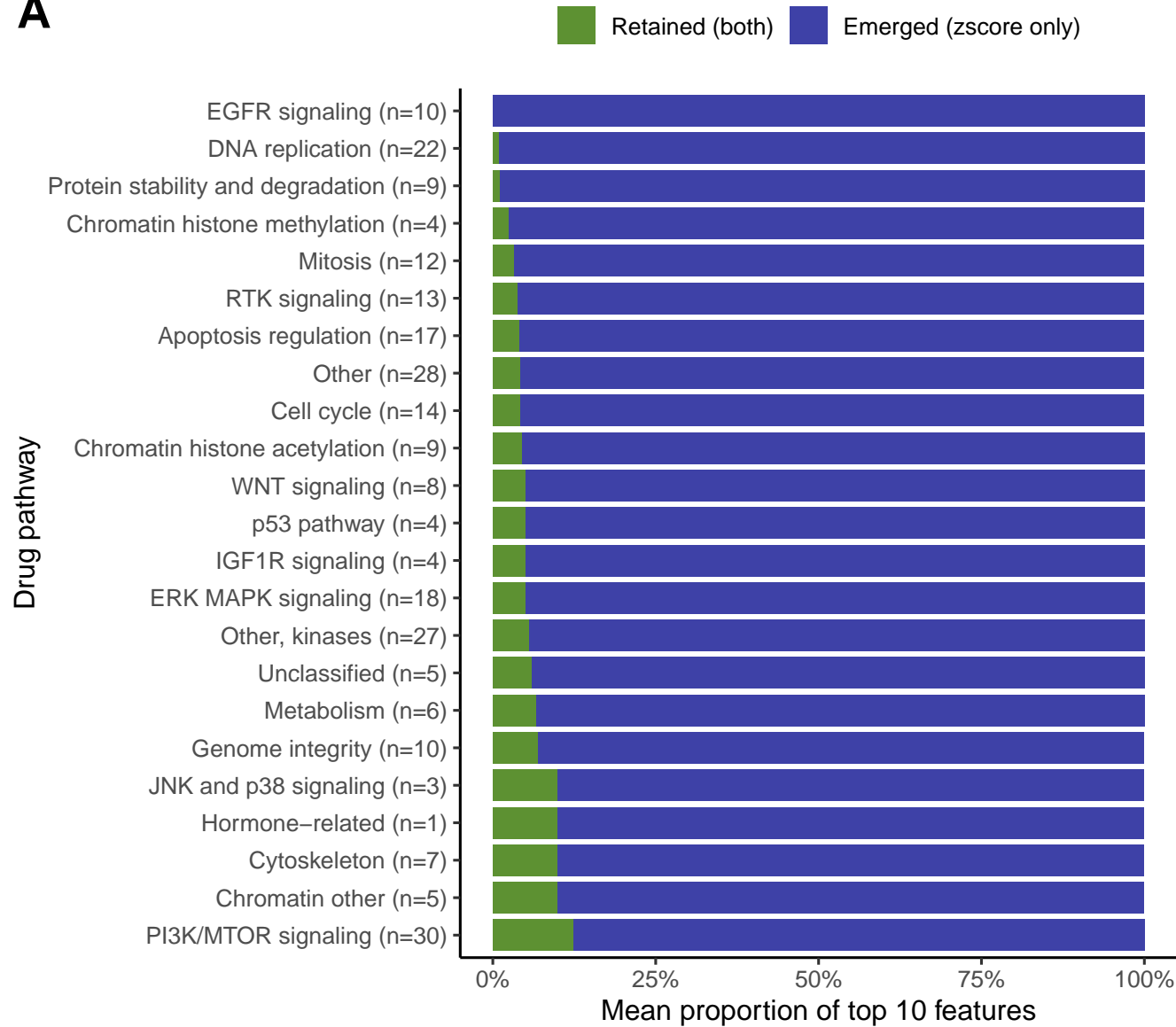**B**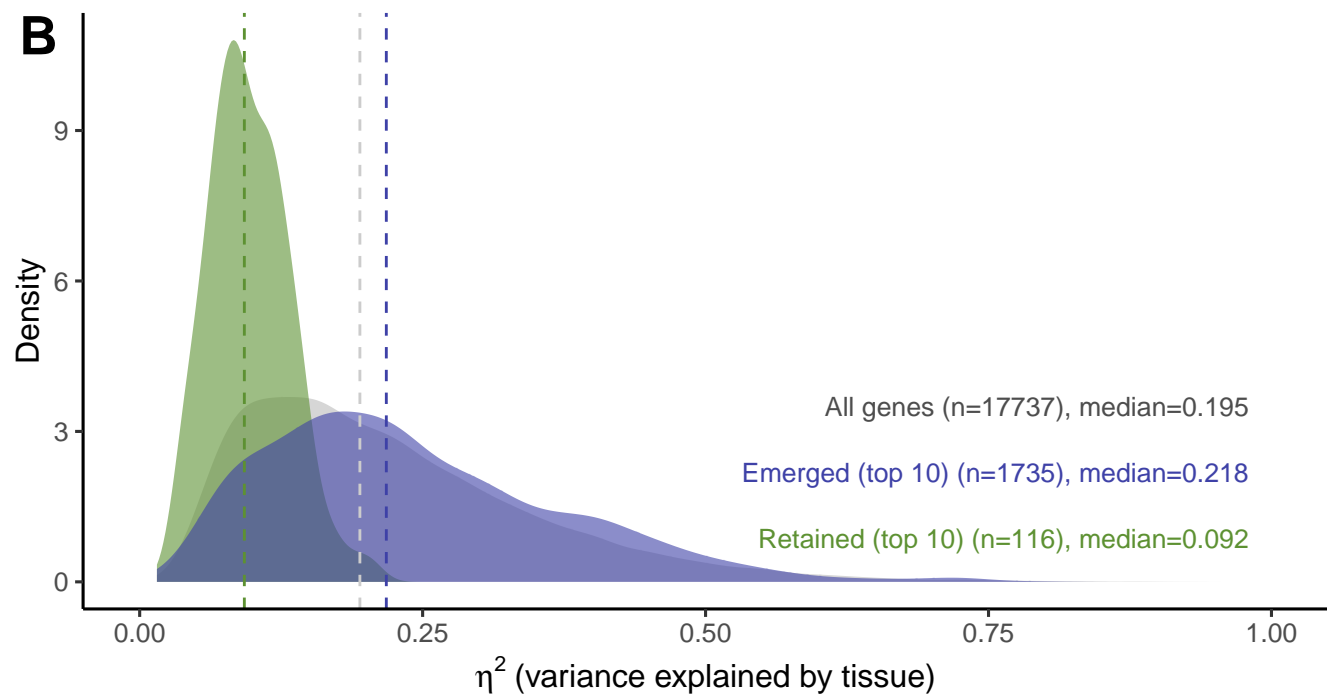**C**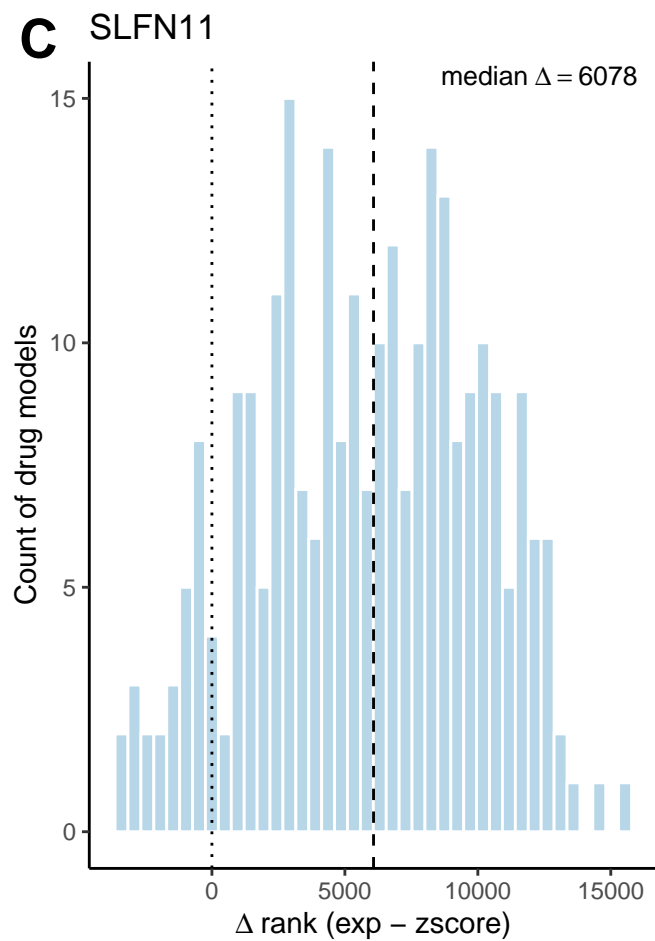**D**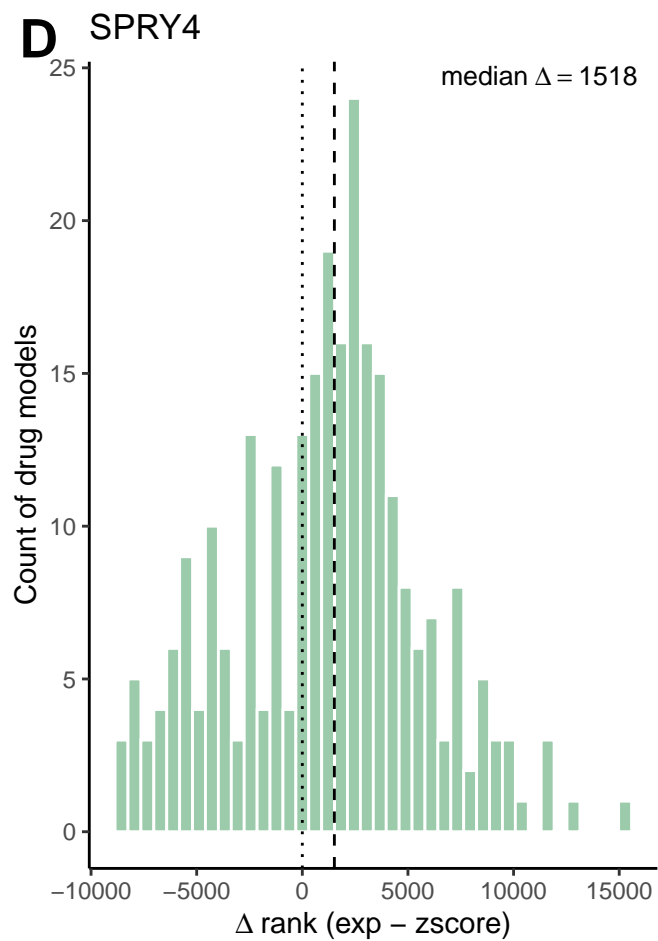**E**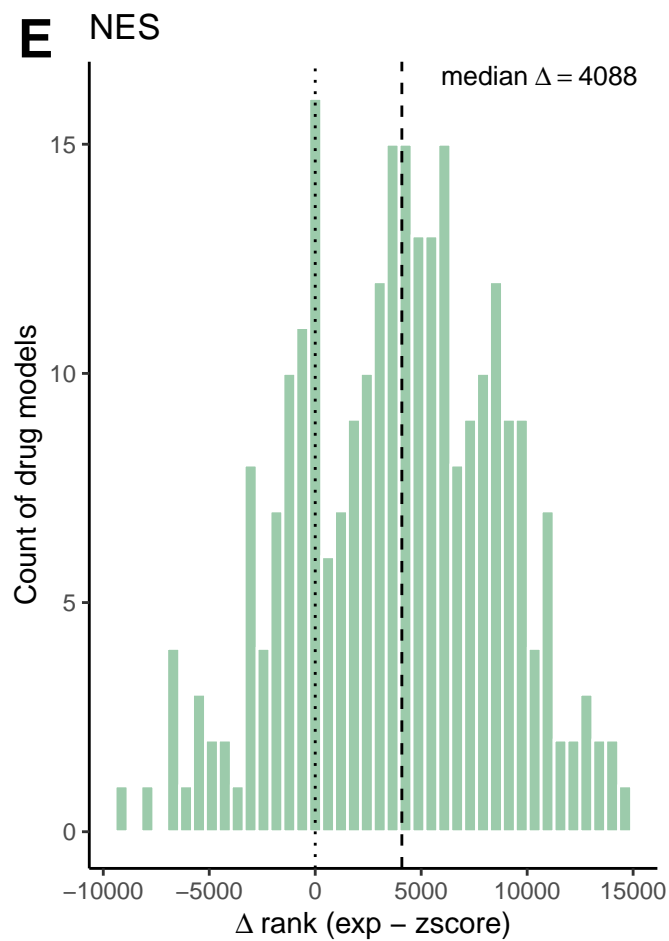**F**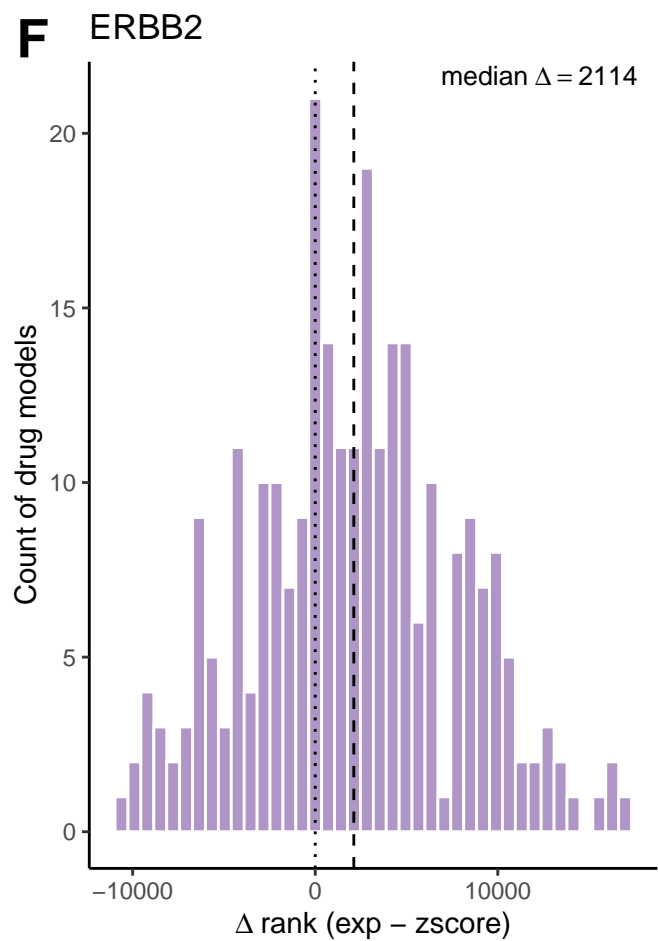

Supplement: S8 Fig — (A) Mean proportion of retained and emerged top 10 gene features per drug pathway; (B) distribution of tissue attribution (η²) across all genes, and for retained and emerged top 10 features; (C) Δrank distribution for SLFN11, (D) SPRY4, (E) NES, and (F) ERBB2 showing rank improvement after tissue correction. (PDF) [file pone.0330412.s016.pdf]

[illegible]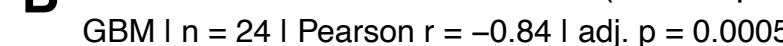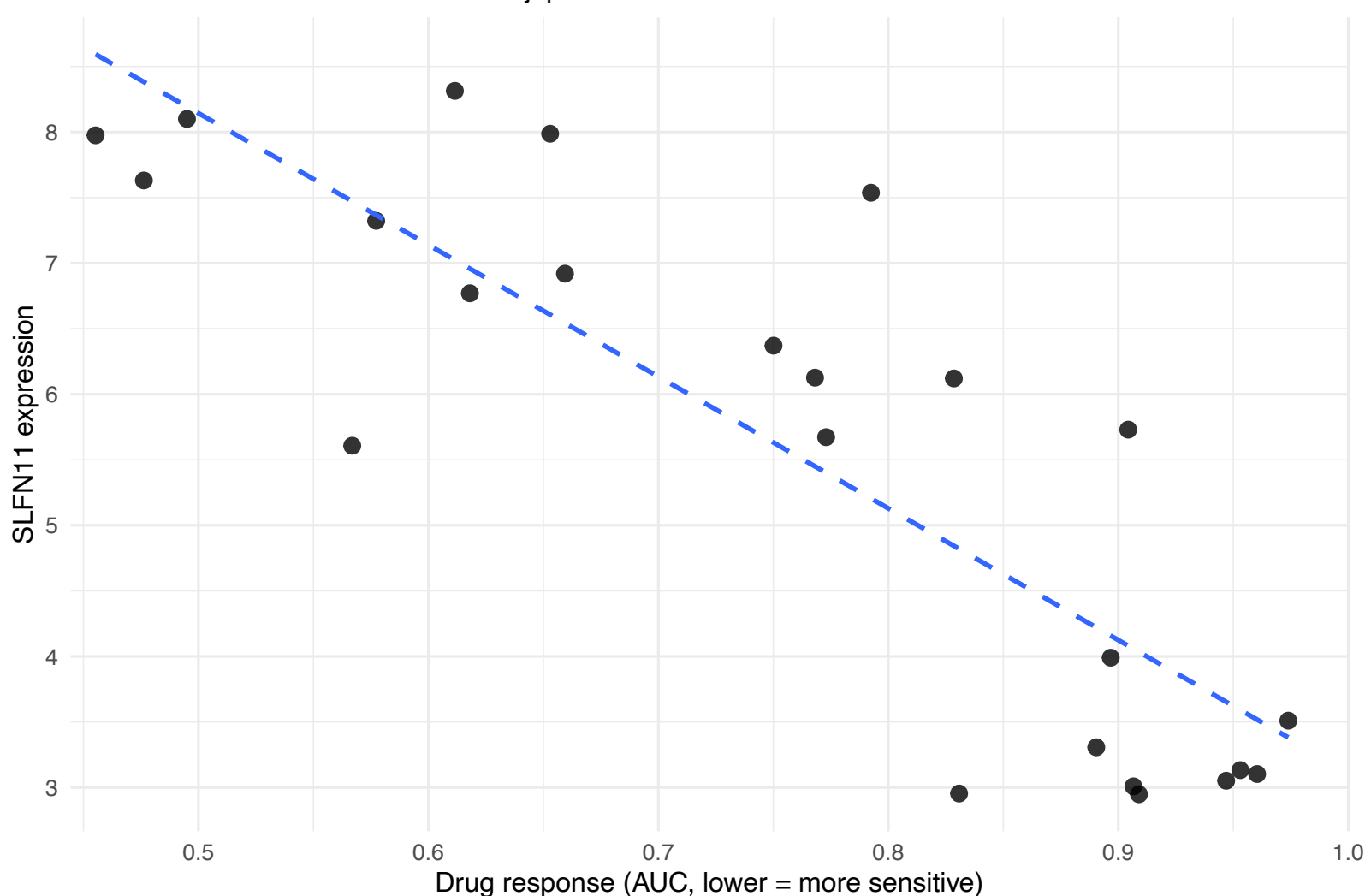

**c** SLFN11 vs Camptothecin\_1003 (DNA replication)

LIHC | n = 16 | Pearson r = -0.83 | adj. p = 0.044

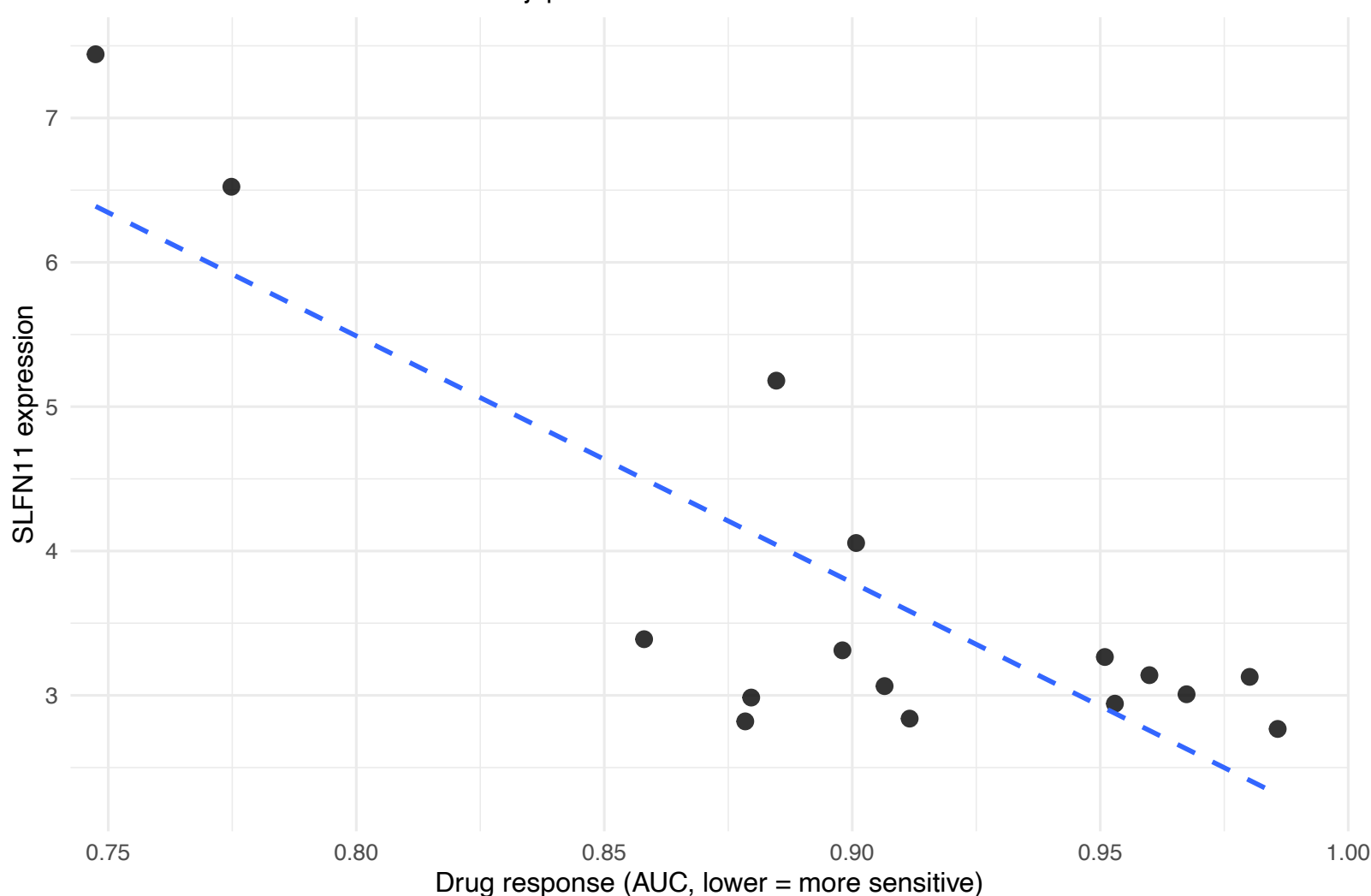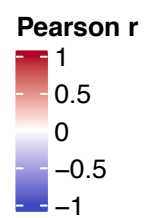

Supplement: S9 Fig — (A) Heatmap of Pearson r between gene expression and drug response (AUC) across cancer types (only drug-type pairs with n ≥ 10 are shown); scatterplots of SLFN11 expression and AUC for (B) gemcitabine (DNA replication) in GMB, and (C) camptothecin (DNA replication) in LIHC. (PDF) [file pone.0330412.s017.pdf]

**Pearson r**

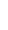

1  
0.5  
0  
-0.5  
-1

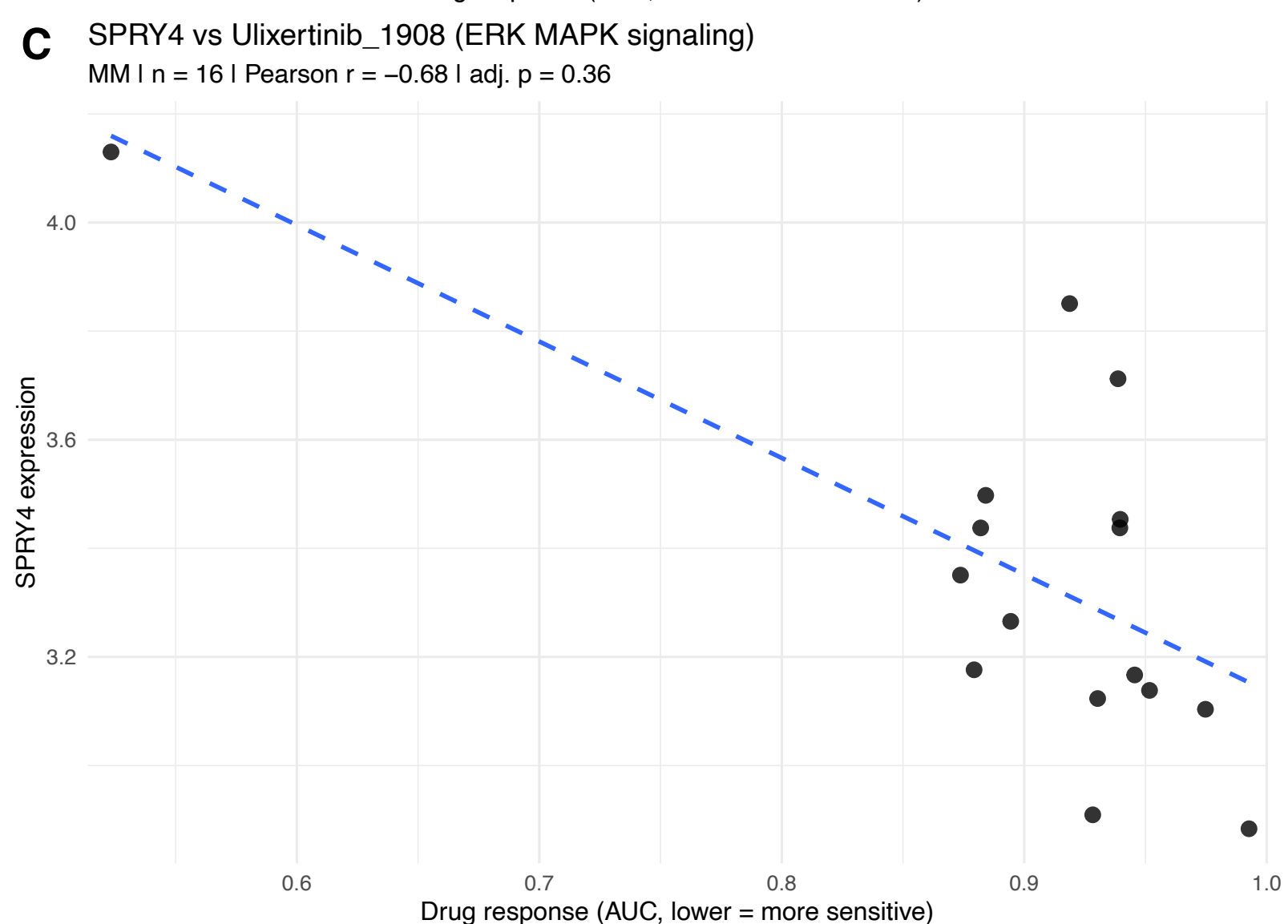

Supplement: S11 Fig — (A) Heatmap of Pearson r between gene expression and drug response (AUC) across cancer types (only drug-type pairs with n ≥ 10 are shown); scatterplots of SPRY4 expression and AUC for (B) AZ628 (ERK MAPK signaling) in BRCA, and (C) ulixertinib (ERK MAPK signaling) in MM. (PDF) [file pone.0330412.s019.pdf]

**Δ**

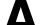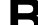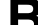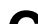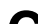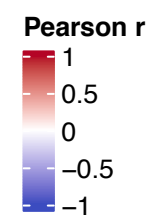

Supplement: S13 Fig — (A) Heatmap of Pearson r between gene expression and drug response (AUC) across cancer types (only drug-type pairs with n ≥ 10 are shown); scatterplots of IVL expression and AUC for (B) gefitinib (EGFR signaling) in CESC, and (C) AZD3759 (EGFR signaling) in CESC. (PDF) [file pone.0330412.s021.pdf]

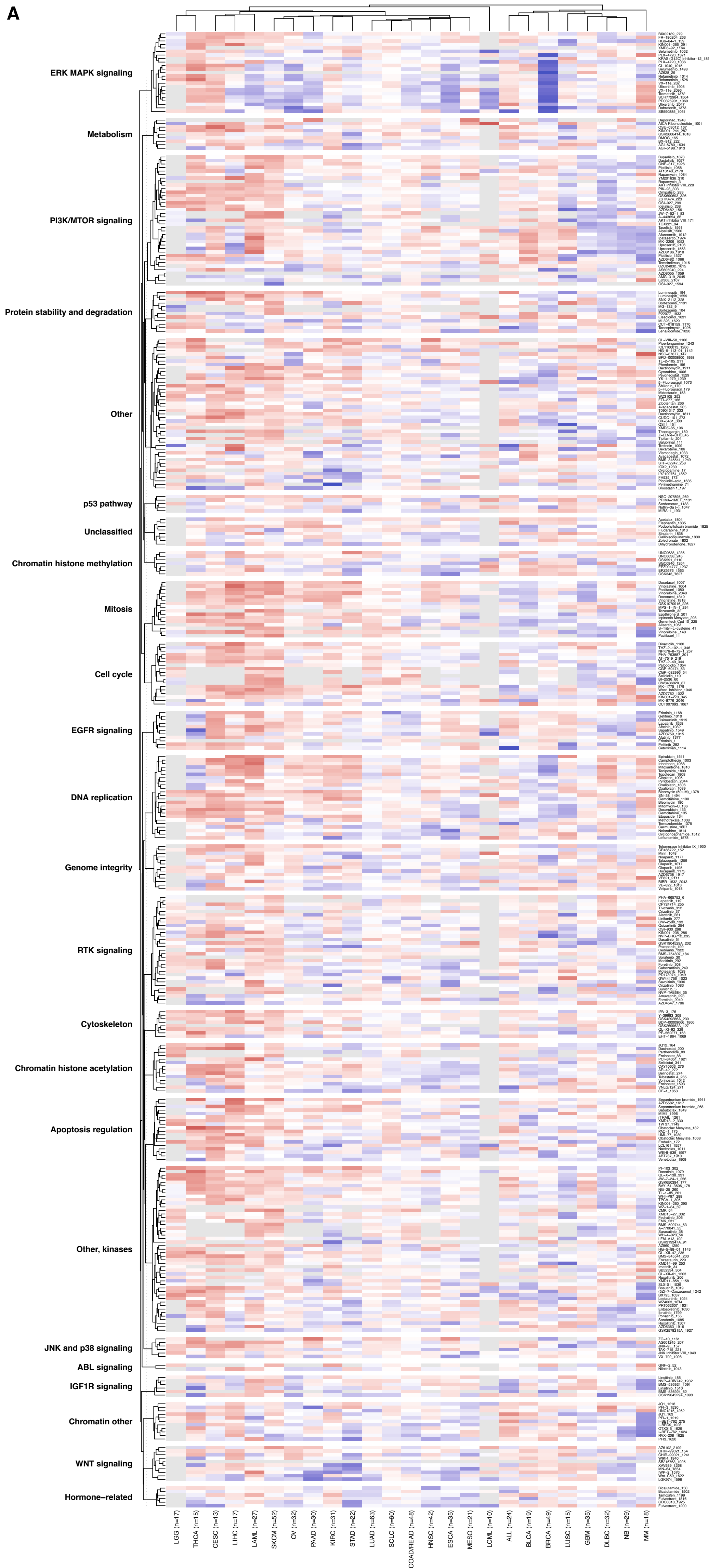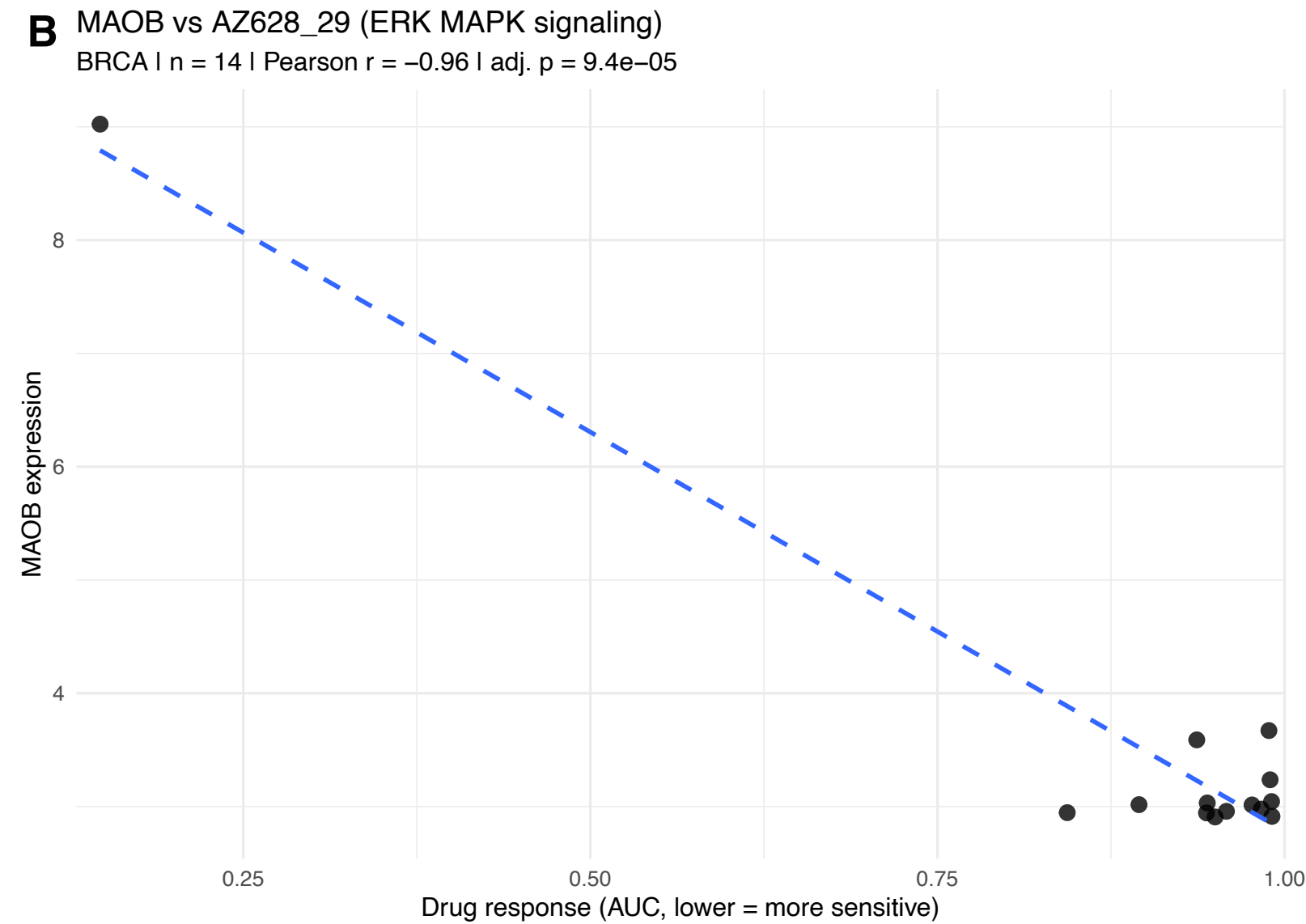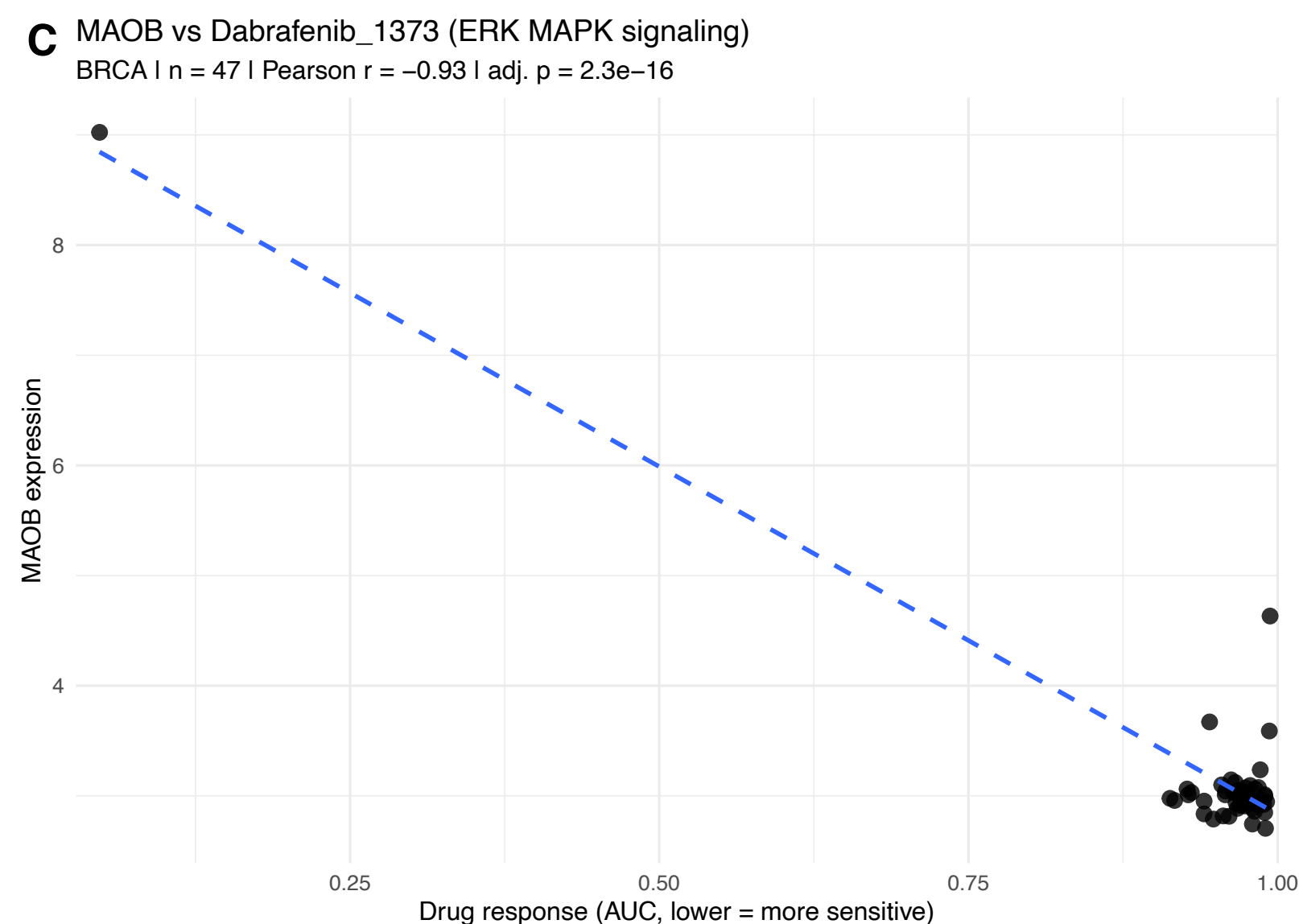

Supplement: S14 Fig — (A) Heatmap of Pearson r between gene expression and drug response (AUC) across cancer types (only drug-type pairs with n ≥ 10 are shown); scatterplots of MAOB expression and AUC for (B) AZ628 (ERK MAPK signaling) in BRCA, and (C) dabrafenib (ERK MAPK signaling) in BRCA. (PDF) [file pone.0330412.s022.pdf]

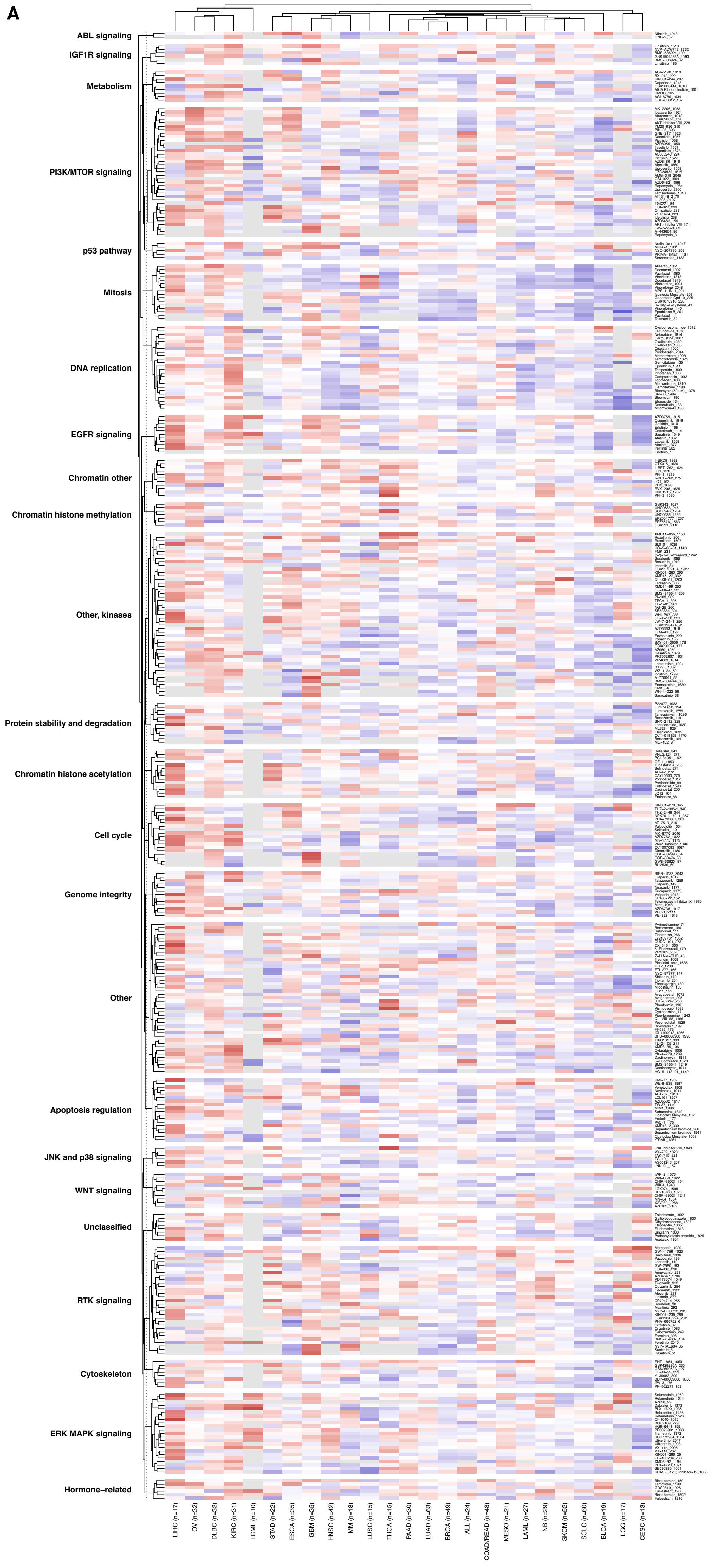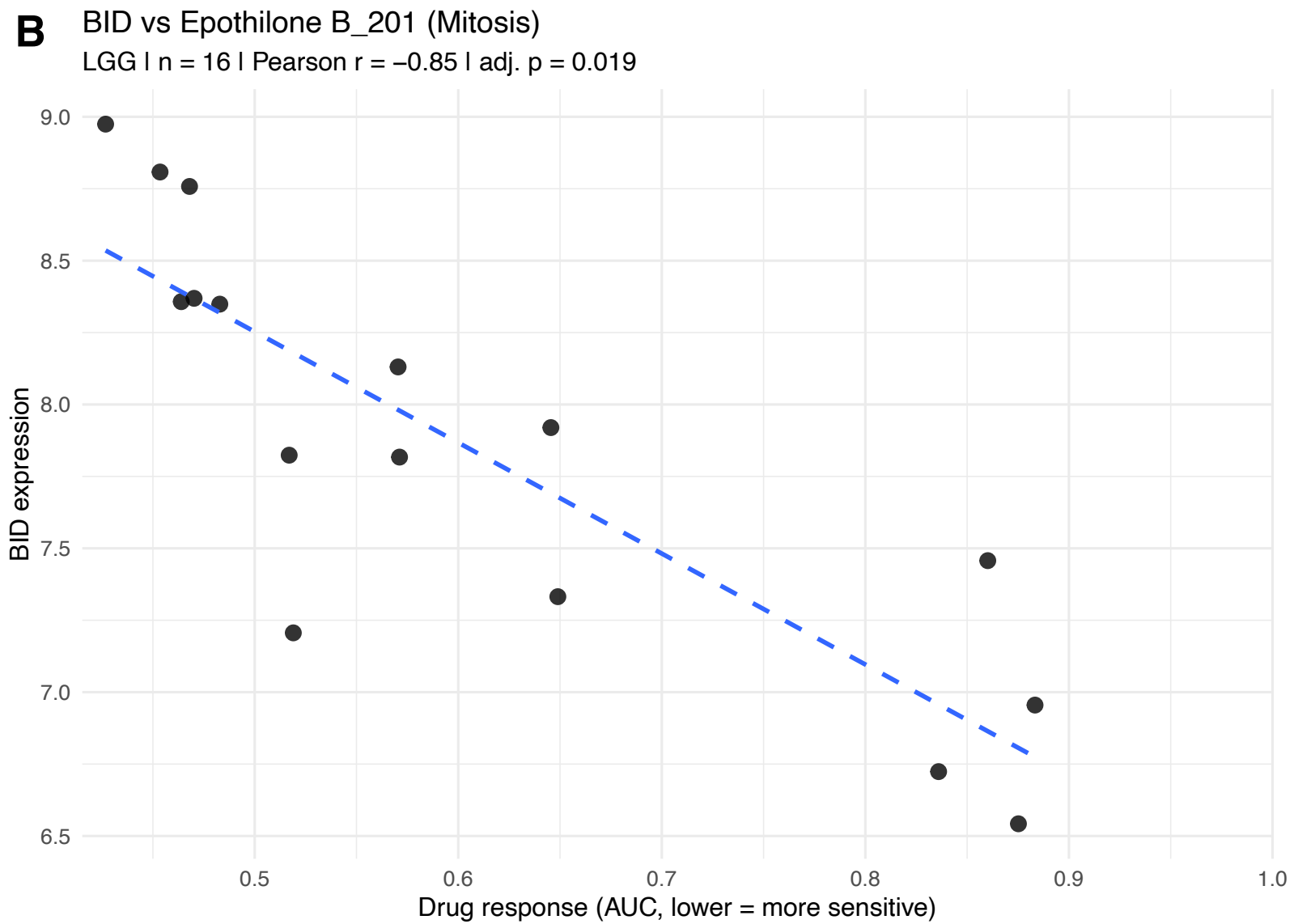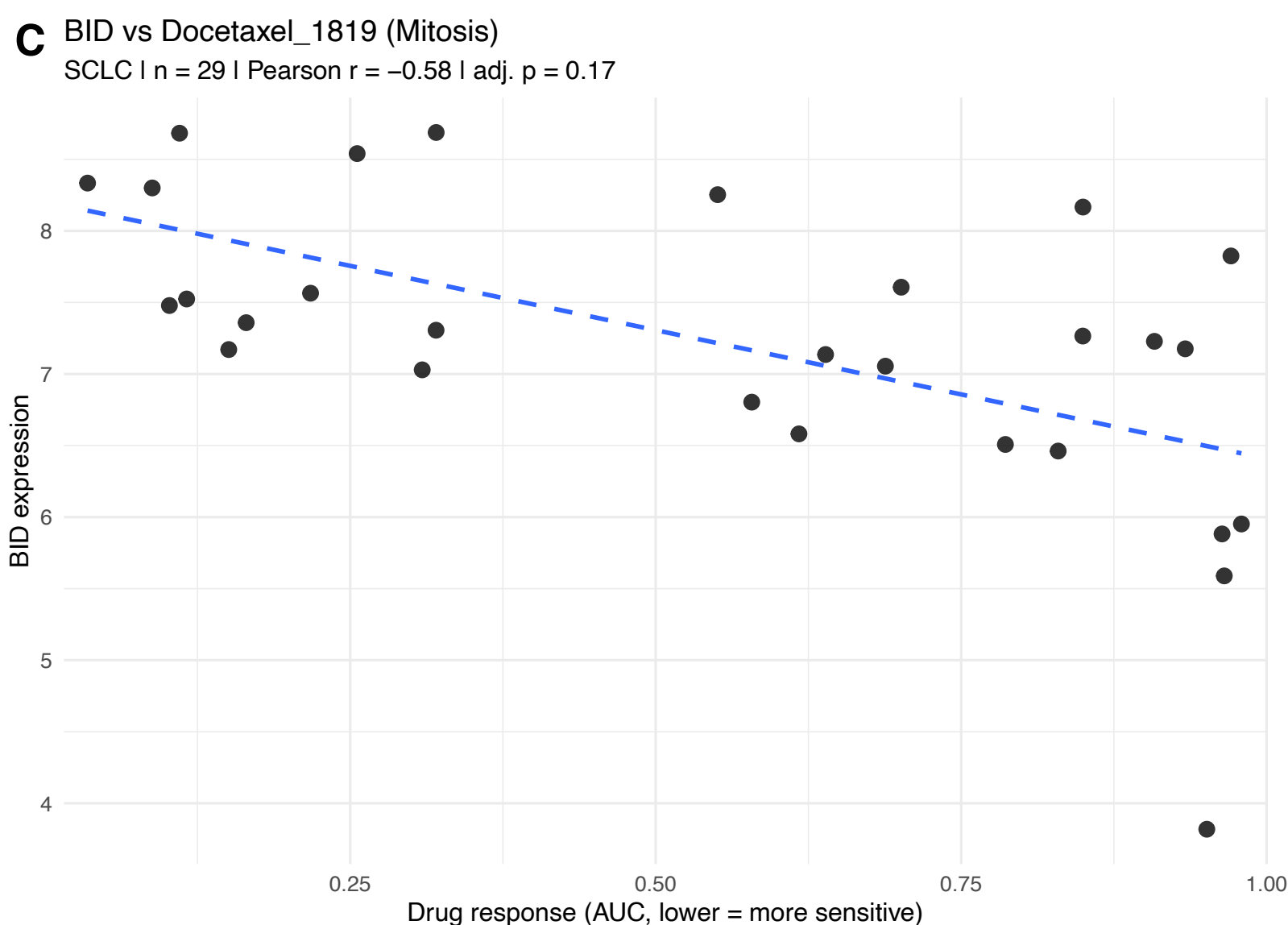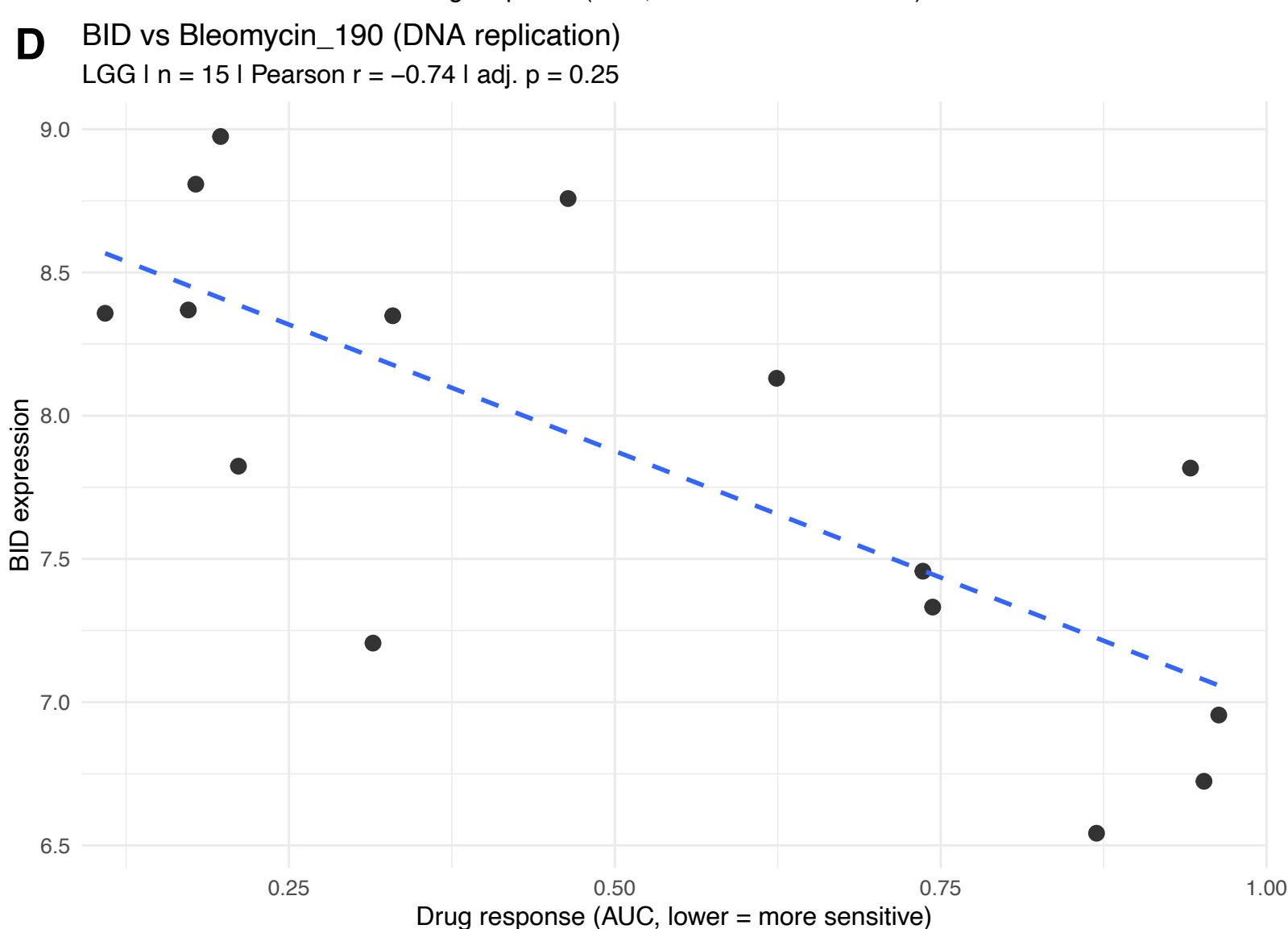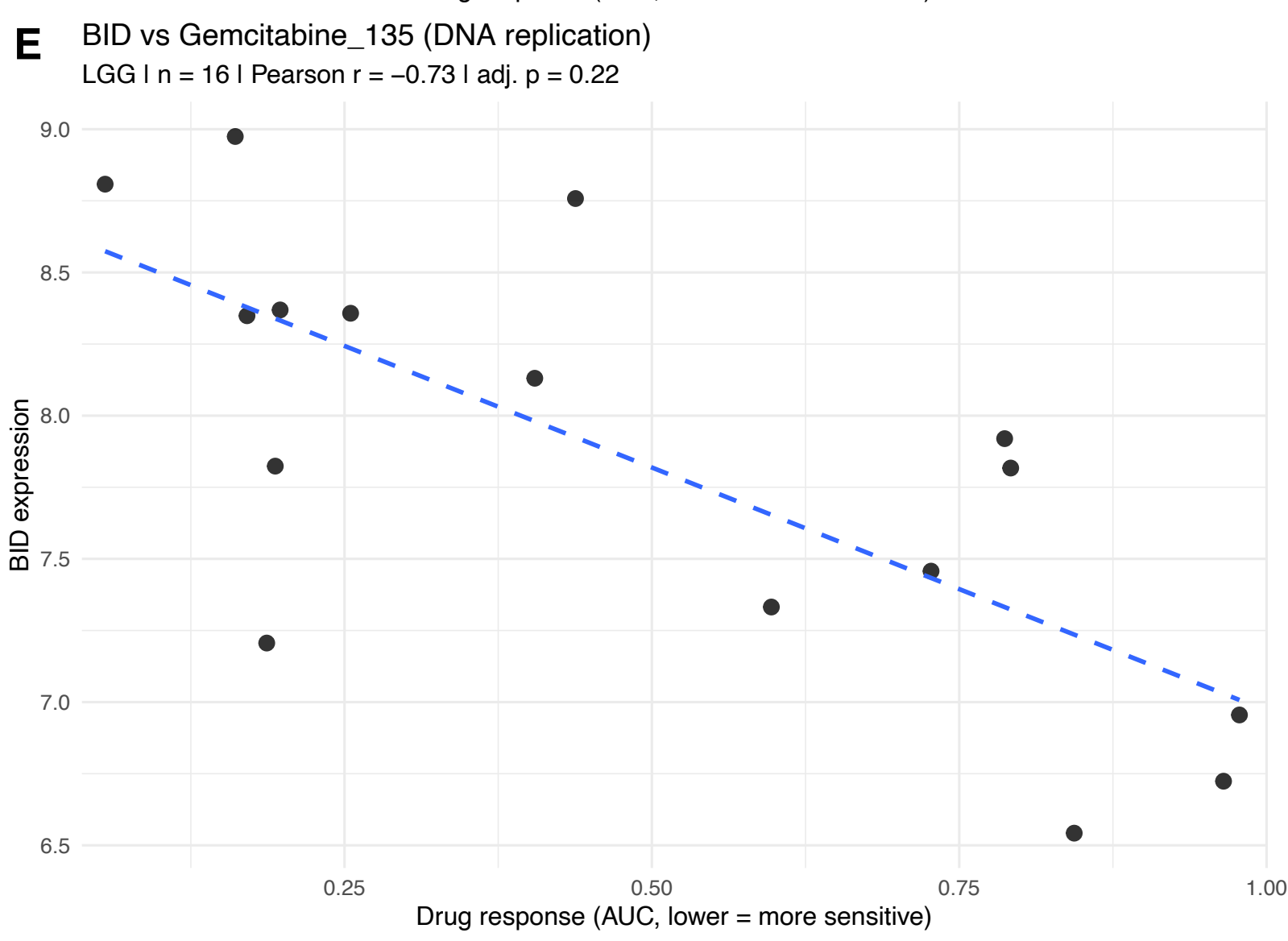

Supplement: S15 Fig — (A) Heatmap of Pearson r between gene expression and drug response (AUC) across cancer types (only drug-type pairs with n ≥ 10 are shown); scatterplots of BID expression and AUC for (B) epothilone B (mitosis) in LGG, (C) docetaxel (mitosis) in SCLC, (D) bleomycin (DNA replication) in LGG, and (E) gemcitabine (DNA replication) in LGG. (PDF) [file pone.0330412.s023.pdf]
