# Supplementary material for: Systematic identification of pan-cancer single-gene expression biomarkers in drug high-throughput screens
Source: PLoS One. 2026 May 11;21(5):e0330412. doi: 10.1371/journal.pone.0330412 (PMC13160354; doi:10.1371/journal.pone.0330412)
Supplement: S10 Fig — (A) Heatmap of Pearson r between gene expression and drug response (AUC) across cancer types (only drug-type pairs with n ≥ 10 are shown); scatterplots of ERBB2 expression and AUC for (B) osimertinib (EGFR signaling) in LUSC, and (C) afatinib (EGFR signaling) in BRCA. (PDF) [file pone.0330412.s018.pdf]

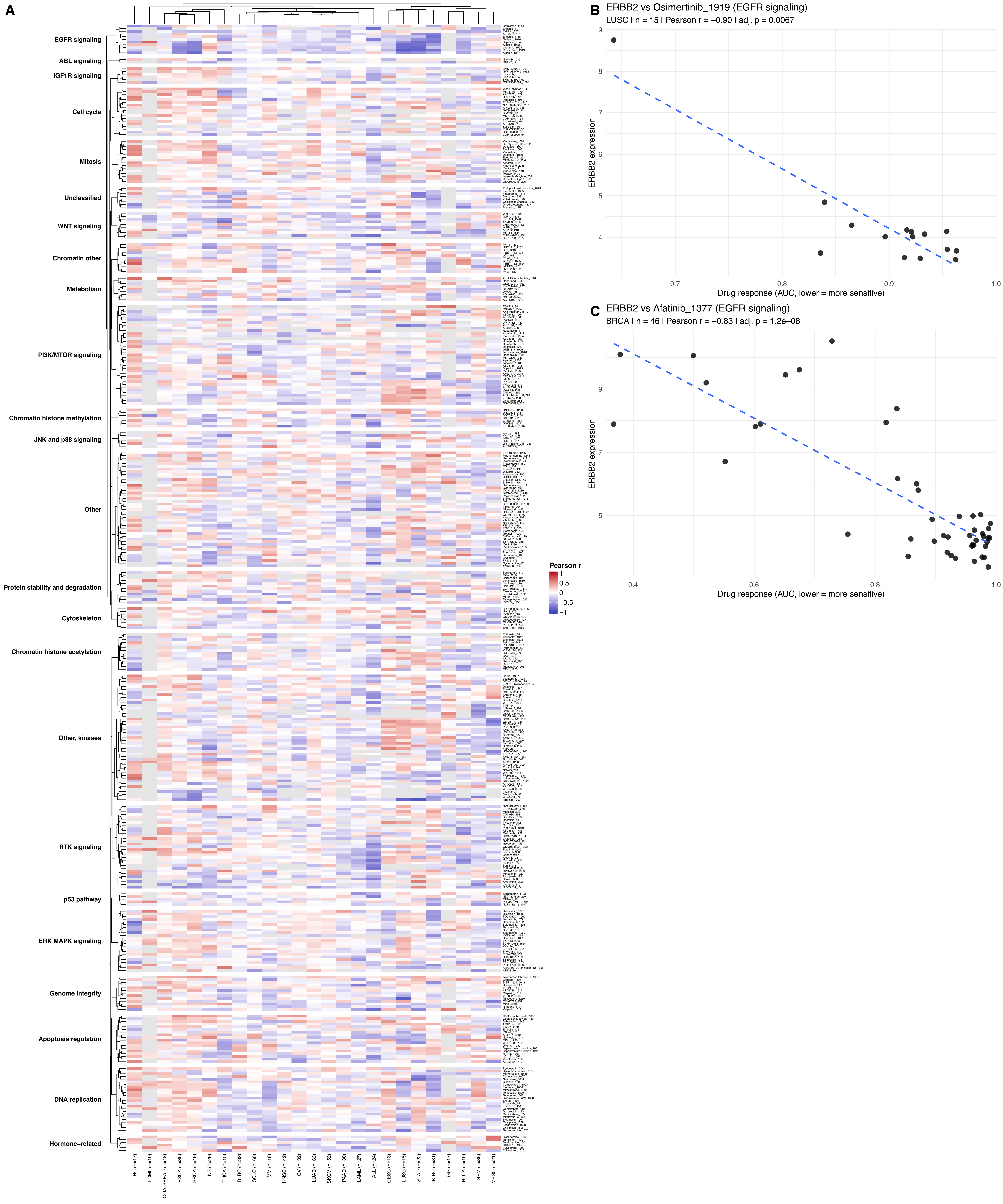
A
EGFR signaling
ABL signaling
IGF1R signaling
Cell cycle
Mitosis
Unclassified
WNT signaling
Chromatin other
Metabolism
PI3K/MTOR signaling
Chromatin histone methylation
JNK and p38 signaling
Other
Protein stability and degradation
Cytoskeleton
Chromatin histone acetylation
Other, kinases
RTK signaling
p53 pathway
ERK MAPK signaling
Genome integrity
Apoptosis regulation
DNA replication
Hormone-related
LIHC (n=17)
LCML (n=10)
COAD/READ (n=48)
ESCA (n=35)
BRCA (n=49)
NB (n=29)
THCA (n=15)
DLBC (n=32)
SCLC (n=60)
MM (n=18)
HNSC (n=42)
OV (n=32)
LUAD (n=63)
SKCM (n=52)
PAAD (n=30)
LAML (n=27)
ALL (n=24)
CESC (n=13)
LUSC (n=15)
STAD (n=22)
KIRC (n=31)
LGG (n=17)
BLCA (n=19)
GBM (n=35)
MESO (n=21)
Pearson r
1
0.5
0
−0.5
−1
B
ERBB2 vs Osimertinib_1919 (EGFR signaling)
LUSC | n = 15 | Pearson r = −0.90 | adj. p = 0.0067
ERBB2 expression
Drug response (AUC, lower = more sensitive)
C
ERBB2 vs Afatinib_1377 (EGFR signaling)
BRCA | n = 46 | Pearson r = −0.83 | adj. p = 1.2e−08
ERBB2 expression
Drug response (AUC, lower = more sensitive)
