# Supplementary material for: Systematic identification of pan-cancer single-gene expression biomarkers in drug high-throughput screens
Source: PLoS One. 2026 May 11;21(5):e0330412. doi: 10.1371/journal.pone.0330412 (PMC13160354; doi:10.1371/journal.pone.0330412)
Supplement: S12 Fig — (A) Heatmap of Pearson r between gene expression and drug response (AUC) across cancer types (only drug-type pairs with n ≥ 10 are shown); scatterplots of NES expression and AUC for (B) VX-11e (ERK MAPK signaling) in BLCA, and (C) PD0325901 (ERK MAPK signaling) in BLCA. (PDF) [file pone.0330412.s020.pdf]

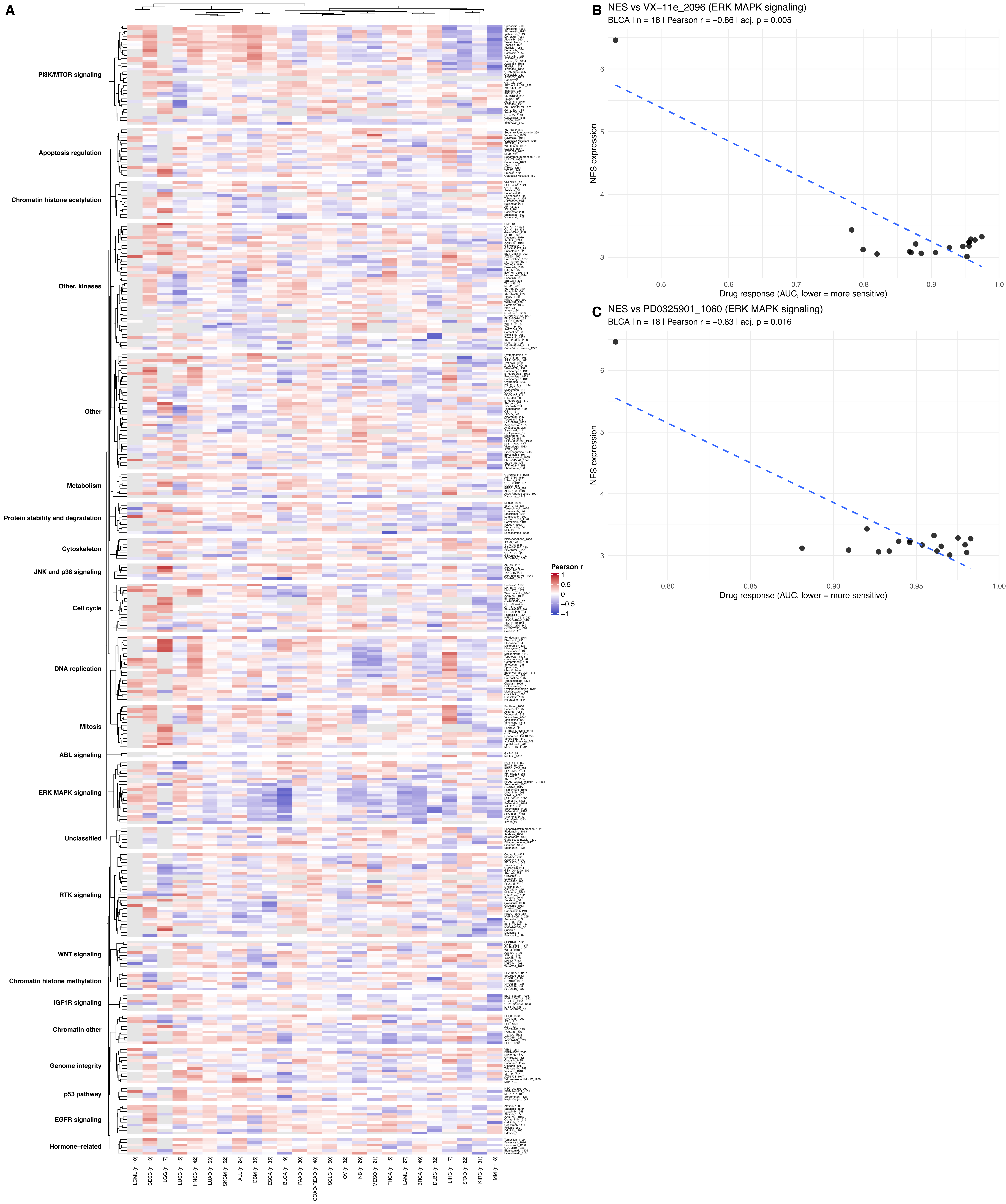
A
PI3K/MTOR signaling
Apoptosis regulation
Chromatin histone acetylation
Other, kinases
Other
Metabolism
Protein stability and degradation
Cytoskeleton
JNK and p38 signaling
Cell cycle
DNA replication
Mitosis
ABL signaling
ERK MAPK signaling
Unclassified
RTK signaling
WNT signaling
Chromatin histone methylation
IGF1R signaling
Chromatin other
Genome integrity
p53 pathway
EGFR signaling
Hormone-related
LCML (n=10)
CESC (n=13)
LGG (n=17)
LUSC (n=15)
HNSC (n=42)
LUAD (n=63)
SKCM (n=52)
ALL (n=24)
GBM (n=35)
ESCA (n=35)
BLCA (n=19)
PAAD (n=30)
COAD/READ (n=48)
SCLC (n=60)
OV (n=32)
NB (n=29)
MESO (n=21)
THCA (n=15)
LAML (n=27)
BRCA (n=49)
DLBC (n=32)
LIHC (n=17)
STAD (n=22)
KIRC (n=31)
MM (n=18)
Pearson r
1
0.5
0
−0.5
−1
B
NES vs VX−11e_2096 (ERK MAPK signaling)
BLCA | n = 18 | Pearson r = −0.86 | adj. p = 0.005
NES expression
Drug response (AUC, lower = more sensitive)
C
NES vs PD0325901_1060 (ERK MAPK signaling)
BLCA | n = 18 | Pearson r = −0.83 | adj. p = 0.016
NES expression
Drug response (AUC, lower = more sensitive)
